# Comparative analysis of IDF, ATPIII and CDS in the diagnosis of metabolic syndrome among adult inhabitants in Jiangxi Province, China

**DOI:** 10.1371/journal.pone.0189046

**Published:** 2017-12-07

**Authors:** Lengmei Cheng, Wei Yan, Liping Zhu, Yiying Chen, Jie Liu, Yan Xu, Lu Ji, Junrong He

**Affiliations:** 1 School of Public Health, Nanchang University, Nanchang, P.R. China; 2 Jiangxi Provincial Center for Disease Control and Prevention, Nanchang, P.R. China; 3 Ganzhou People’s Hospital, Ganzhou, P.R. China; Shanghai Diabetes Institute, CHINA

## Abstract

**Background:**

Currently, the prevalence of metabolic syndrome (MS) has attracted widespread public attention. However, there is a war regarding the applicability of the diagnosis in different populations regarding the distinct criteria for the diagnosis of MS. Data about the prevalence rate of MS and its components in Jiangxi Province are limited. Thus, our goals were to compare the consistency rates and applicability of three criteria, i.e., those of the International Diabetes Federation (IDF), the National Cholesterol Education Program Adult Treatment PanelⅢ (ATPⅢ), and the Chinese Diabetes Society (CDS).

**Methods:**

From September 2013 to March 2014, 5959 residents (age≥18 years) from Jiangxi Province were selected by multistage stratified cluster random sampling methods. The prevalence rate of MS and its components were calculated according to the IDF, ATPⅢ and CDS criteria, and the protocols of the different criteria were measured in terms of consistency with the kappa statistic and Youden’s index. Receiver operator characteristic (ROC) curve analysis was used to explore the optimal cut-off points of body mass index (BMI) and waist circumference (WC).

**Results:**

The mean age of the participants was 50.52±13.92 years among the total of 5959 individuals (2451 male and 3508 female). The standardized prevalence rate of MS was 19.85%, 24.77% and 9.95% according to the IDF, ATPⅢ and CDS criteria, respectively. The order of the prevalence rates of the different components of MS according to the IDF or ATPⅢ criteria from high to low were as follows: elevated blood pressure, central obesity, reduced high density lipoprotein cholesterol (HDL-C), hyperglycemia, and hypertriglyceridemia. The most prevalent component of MS, according to the CDS criterion, was being overweight or obese, followed by elevated blood pressure, dyslipidemia and hyperglycemia. The Youden’s index in IDF criterion was higher than which in CDS criterion (0.79 for IDF vs. 0.38 for CDS) referring to the ATPⅢ criterion. The agreement between the IDF and ATPⅢ criteria was good (kappa = 0.85), whereas the agreement of the CDS with the IDF and ATPⅢ criteria was moderate (kappa = 0.46 and 0.46, respectively). The ability to predict MS risk factors clusters was superior when the BMI cut-off point was 24/24kg/m^2^ (male/female), and the WC cut-off point was 87/80cm (male/female). Among the 18~59 years old male group, BMI was superior to WC in predicting clusters of risk factors for MS; while in the 60 years and above male group and all-age female group, WC was superior to BMI.

**Conclusion:**

Our results revealed that the prevalence rate of metabolic syndrome among adults was high in Jiangxi Province. BMI and WC had different ability to predict clusters of risk factors for MS in different age groups and gender. Among the three criteria for MS, there was bigger difference in applicability for the adults of Jiangxi Province. The choice of the appropriate criteria should be based on the actual conditions of the site.

## 1 Introduction

MS is a clinical condition that includes multiple cardiovascular disease risk factors, such as obesity, elevated blood pressure or hypertension, dyslipidemia, and abnormal glucose metabolism, which directly increase the risk of coronary heart disease, atherosclerotic cardiovascular disease and type 2 diabetes[1, 2].

However, thus far, the criteria for MS have not yet been fully accepted, but the International Diabetes Federation (IDF)[3] and the National Cholesterol Education Program Adult Treatment Panel Ⅲ (ATPⅢ)[4] criteria appear to be visible everywhere. Moreover, the Chinese Diabetes Society (CDS)[5]criteria are based on similar components, although they differ in some specific elements and have been investigated by many researchers in China. The contents of three criteria were different, resulting a different prevalence rate when using different criteria in the same population.

Currently, the population is aging, and physical obesity and a lack of exercise are increasing, and these factors have led to a high prevalence of MS worldwide. The prevalence rate of adult MS was 28.9% (ATPⅢ) in Korea[6] from 2008 and 2013. The prevalence rates of MS were 24.3% (ATPⅢ) and 39% (IDF) in 10 European countries[7] and Saudi Arabia[8]. Similarly, the situation in China is not optimistic. A national survey[9] reported that the prevalence rate of MS among the Chinese adults was 18.2% (IDF), 21.3% (ATPⅢ) and 10.5% (CDS), respectively.

In our research, the prevalence rate of MS and its components were analyzed, and the consistency and applicability were compared between the three criteria (i.e., the IDF, ATPⅢ, and CDS criteria) to provide a basis for interventions in Jiangxi Province.

## 2 Materials and methods

### 2.1 Study population

Jiangxi province is located in southeastern China and covers an area of 16.6 million square kilometers, a total of 11 cities, and 100 counties. The population is 45,000,000[10]. From September 2013 to March 2014, 10 representative disease-monitoring points were identified according to the regional distribution characteristics of Jiangxi Province, and 5959 residents aged over 18 years old were selected by multistage stratified cluster random sampling methods from 10 disease surveillance sites. First, we randomly selected four townships from each surveillance district/county using the method of probability proportional to size. Second, three villages or residential areas were then selected from each chosen township by using the same method as in the previous stage. Subsequently, a residential group (at least 50 families) was selected from each chosen village or residential area by simple random sampling. Finally, individuals of at least 18 years of age were selected from each family via a Kish grid. All individuals from Hong Kong, Macao or Taiwan, deaf-mutes, and pregnant female were excluded. The actual number of respondents was 5,959from among the 6,000 investigated people because the effective response rate was 99.32%. After signing the informed consent form, all individuals who met the survey criteria were included, and this study was authorized by the Jiangxi Province Ethics Review Board.

### 2.2 Measures

The research consisted of three parts: questionnaires, physical examinations, and laboratory tests. The field staff who were responsible for the investigations were trained and qualified. The standardized questionnaire survey included name, sex, age, health status, and diagnoses and treatments for three diseases, including diabetes, hypertension and dyslipidemia. The BMI was calculated as the weight (kg) divided by the square of the height (m). The heights and weights were measured with the individuals wearing light clothing and without shoes, hats and coats. The waists were measured by the same staff at the level of the umbilical cord while the individuals were standing and responding normally. After 5 minutes of sitting, all of the individuals' blood pressures were measured three times with an electronic arm sphygmomanometer (HEM-7071, Omron Healthcare, Inc., Kyoto, Japan) with a measurement interval of 1 minute. The average of the last two measurements was used for the analyses. To measure the fasting plasma glucose and the 2-hour postprandial plasma glucose, venous blood samples were drawn from everyone, and the fasted overnight plasma glucose was measured at the local monitoring site. The samples were centrifuged at 3000 rpm for 5 to 10 minutes. To measure the blood lipids, the sera were extracted within 2 hours of collection and transported on dry ice to the national laboratory in Shanghai Ruijin Hospital. IDF, ATPⅢ and CDS criteria were employed to estimate the prevalence of MS in Jiangxi province to compare them with other populations. These three criteria are presented in Table 1.

**Table 1:** The three criteria for MS.

Components	IDF (2005)	ATPⅢ(2005)	CDS (2004)
Obesity (Chinese)	waist circumference (WC)≥90cm (male) or ≥80 cm (female)	1.waist circumference (WC)≥90 cm (male) or ≥80 cm (female)	1.BMI≥25.0 kg/m^2^
Elevated blood pressure	1.systolic blood pressure (SBP)≥130 or diastolic blood pressure (DBP) ≥85 mmHg or treatment of previously diagnosed hypertension	2.systolic blood pressure (SBP)≥130 or diastolic blood pressure (DBP)≥85 mmHg or on antihypertensive drug treatment in a patient with a history of hypertension	2.systolic blood pressure (SBP)≥140ordiastolic blood pressure (DBP)≥90mmHg and (or) treatment of previously diagnosed hypertension
Hyperglycemia	2.fasting plasma glucose (FPG) ≥ 100 mg/dL (5.6) mmol/L), or previously diagnosed type 2 diabetes	3.fasting plasma glucose (FPG)≥100 mg/dL (5.6mmol/L) or on drug treatment for elevated glucose	3.fasting plasma glucose (FPG)≥110 mg/dL (6.1 mmol/L) and (or) 2 hours postprandial plasma glucose (2hPPG)≥140 mg/dL (7.8 mmol/L), and (or) on drug treatment for diabetes
Dyslipidemia	3.triglyceride (TG)≥ 150 mg/dL (1.7 mmol/L) orspecific treatment for this lipid abnormality at high-density4.high density lipoprotein cholesterol (HDL-C)< 40 mg/dL (1.03 mmol/L) (male),<50 mg/dL (1.29 mmol/L)(female)or specific treatment for this lipid abnormality	4.triglyceride (TG)≥ 150 mg/dL (1.7 mmol/L) or on drug treatment for elevated triglycerides 5.high density lipoprotein cholesterol (HDL-C)< 40 mg/dL (1.03 mmol/L) (male),<50 mg/dL (1.3 mmol/L) (female)or on drug treatment for reduced HDL	4.triglyceride(TG)≥ 150 mg/dL (1.7 mmol/L) and (or) high density lipoprotein cholesterol (HDL-C)< 35 mg/dL (0.9 mmol/L) (male)< 39 mg/dL (1.0 mmol/L) (female)

Note: MS was diagnosed as follows: the IDF criterion: a person with abdominal obesity (waist circumference≥90 cm (male) or ≥80 cm (female), plus any two risk factors labeled 1~4; the ATPⅢ criterion: a person with at least three of the factors labeled 1~5; and the CDS criterion: a person with at least three of the factors labeled 1~4.

### 2.3 Statistical analysis

The Statistical Product and Service Solutions version 21.0 statistical software was used for the statistical analysis. The variables that followed the normal distribution and were continuous are reported as the means ± the standard deviations (SDs) and were compared with Student's t tests. The categorical variables are reported as percentages and were compared with Pearson’s chi-square tests. All *p* values presented are based on two-tailed tests, and *p*< 0.05 was considered statistically significant. The ROC curve according to the formula for the distance of the ROC curve ($\sqrt{{\left(1-\mathrm{specificity}\right)}^{2}+{\left(1-\mathrm{sensitivity}\right)}^{2}}$)[11]. A cut-off point with the shortest distance of the ROC curve was defined as the optimal cut-off point to diagnose MS. The official 2010 census data of China was used to calculate the age-standardized rates[12].

## 3 Results

### 3.1 Characteristics of the research participants

Five thousand nine hundred fifty-nine individuals, including 2451 male and 3508 female were sampled. The differences in age, WC, height, weight, SBP, DBP, TG, and HDL-C between the male and female were statistically significant (P<0.05). The ages, heights, weights, WCs, SBPs, DBPs and TGs of male were all higher than those of the female, whereas the HDL-Cs were lower among the male. The differences in the BMIs, FPGs, 2hPPGs, LDL-C and CHOL were not statistically significant (P>0.05) (Table 2).

**Table 2:** Characteristics of the participants.

Variables	Total	Male	Female	*P*
N	5959	2451	3508	—
Age (years)	50.52±13.92	51.23±14.47	50.03±13.50	0.01*
Height(cm)	157.42±8.12	163.44±6.82	153.22±6.04	<0.01*
Weight(kg)	57.95±10.48	62.56±10.89	54.74±8.86	<0.01*
WC (cm)	80.80±9.33	82.73±9.52	79.46±8.96	<0.01*
BMI (kg/m^2^)	23.31±3.32	23.34±3.33	23.28±3.31	0.48*
SBP (mmHg)	127.42±20.09	129.65±18.78	125.87±20.81	<0.01*
DBP (mmHg)	75.42±11.12	77.61±11.43	73.88±10.63	<0.01*
FPG (mmol/L)	5.52±1.33	5.56±1.32	5.49±1.34	0.05*
2hPPG (mmol/L)	6.22±2.35	6.18±2.53	6.25±2.22	0.32*
HDL-C (mmol/L)	1.37±0.38	1.33±0.40	1.40±0.36	<0.01*
LDL-C(mmol/L)	2.86±0.83	2.85±0.82	2.87±0.83	0.33*
CHOL(mmol/L)	4.66±0.93	4.65±0.92	4.67±0.94	0.42*
TG (mmol/L)	1.40±1.16	1.49±1.36	1.33±0.99	<0.01*

* Compared between male and female

The data are presented as the means ± the SDs.

WC, waist circumference; BMI, body mass index; SBP, systolic blood pressure; DBP, diastolic blood pressure; FPG, fasting plasma glucose; 2hPPG, 2-hour postprandial plasma glucose; HDL-C, high-density lipoprotein cholesterol; LDL-C, low-density lipoprotein cholesterol; CHOL, cholesterol; TG, triglycerides.

### 3.2 Prevalence rate of MS and its components

The prevalence rate of MS and its components, based on the three different diagnostic criteria, varied widely. The overall prevalence rate of MS was 22.27%, 28.02% and 11.71% according to the IDF, ATPⅢ and CDS criteria, respectively, and after adjusting for age, the standardized prevalence rate was 19.85%, 24.77% and 9.95%, respectively. The prevalence rate of MS was higher in the female than in the male based on the IDF criterion (16.81% for male vs. 26.08% for female) and the ATPⅢ criterion (24.24% for male vs. 30.67% for female) criteria, but this sex difference was reversed when the CDS criterion was employed (13.06% for male vs. 10.78% for female). Elevated blood pressure (41.67%) was the most common metabolic abnormality among the components of MS followed by central obesity (37.74%), reduced HDL-C (34.75%), hyperglycemia (32.19%), and hypertriglyceridemia (24.03%) according to the IDF criterion. The components according to the ATPⅢ criterion also followed the same trend as the IDF criterion. The prevalence rates of central obesity, reduced HDL-C, and MS in female were higher than those in the male (*P*<0.05), whereas the prevalence rates of hypertriglyceridemia, elevated blood pressure, and hyperglycemia in the male were higher than in the female (*P*<0.05) according to the IDF and ATPⅢ criteria. Using the CDS criterion, the most prevalent component of MS was overweight or obesity (28.28%), followed by elevated blood pressure (27.79%), dyslipidemia (25.57%), and hyperglycemia (22.59%). The prevalence rates of overweight or obesity, hypertriglyceridemia, elevated blood pressure, hyperglycemia and MS were higher in the male than in the female (*P*<0.05; Table 3).

**Table 3:** The prevalent rates of the components of MS by gender (according to the IDF, ATPⅢ, and CDS criteria).

	Diagnostic criteria	Total (5959)		Male (2451)		Female (3508)		χ^2^	*P*
		Prevalence	Prevalence rate	Prevalence	Prevalence rate	Prevalence	Prevalence rate		
Overweight or obese	CDS	1685	28.28%	747	30.48%	938	26.74%	9.89	<0.01*
Central obesity	IDF/ ATPⅢ	2249	37.74%	609	24.85%	1640	46.75%	294.01	<0.01*
Hypertriglyceridemia	IDF/ ATPⅢ	1432	24.03%	655	26.72%	777	22.15%	16.48	<0.01*
Reduced HDL-C	IDF	2071	34.75%	618	25.21%	1453	41.42%	167.62	<0.01*
	ATPⅢ	2117	35.53%	618	25.21%	1499	42.73%	193.88	<0.01*
Elevated blood pressure	IDF/ ATPⅢ	2483	41.67%	1154	47.08%	1329	37.88%	51.26	<0.01*
	CDS	1656	27.79%	744	30.35%	912	26.00%	14.01	<0.01*
Hyperglycemia	IDF	1918	32.19%	834	34.03%	1084	30.90%	6.31	0.01*
	ATPⅢ	1912	32.09%	833	33.99%	1079	30.76%	6.74	0.01*
	CDS	1346	22.59%	591	24.11%	755	21.52%	5.73	0.02*
Dyslipidemia	CDS	1524	25.57%	670	27.34%	854	24.34%	6.74	0.01*
MS	IDF	1327	22.27%	412	16.81%	915	26.08%	71.69	<0.01*
	ATPⅢ	1670	28.02%	594	24.24%	1076	30.67%	29.65	<0.01*
	CDS	698	11.71%	320	13.06%	378	10.78%	7.26	0.01*
MS**	IDF	—	19.85%	—	16.98%	—	22.11%	—	—
	ATPⅢ	—	24.77%	—	23.86%	—	25.76%	—	—
	CDS	—	9.95%	—	12.31%	—	8.50%	—	—

* Compared between the male and female.

** Standardized prevalence rate of MS.

### 3.3 Comparison of authenticity and consistency among different criteria

There were 1720 cases was detected among 5959 individuals using the IDF, ATPⅢ and CDS criteria. The overall prevalence rate of MS was 28.86%. The prevalence rate was 9.25%, 14.65%, 4.97% separately when three kinds criteria, two kinds criteria and only one criterion were used at the same time. This indicated there was a large difference among the application of these criteria. Compare the authenticity of IDF and CDS criteria referring the ATPⅢ criterion (Table 4). The results showed the IDF criterion sensitivity was 79.40% (male 69.36%, female 85.94%) and the specificity was 99.98% (male 100%, female 99.96%) in the diagnosis of MS, and the CDS criterion sensitivity was 38.86% (male 48.48%, female 33.55%), the specificity was 98.86% (male 98.28%, female 99.30%) in the diagnosis of MS. The Youden’s index was higher in IDF criterion than in CDS criterion (0.79 for IDF vs. 0.38 for CDS) which indicated it was more superior to distinguish the true MS patients and non-patients by using IDF criterion than CDS criterion.

**Table 4:** Analysis on validity of different criteria for MS.

Test criteria	Reference criteria	Total			Male			Female		
		Sensitivity (%)	Specificity (%)	Youden’s index	Sensitivity (%)	Specificity (%)	Youden’s index	Sensitivity (%)	Specificity (%)	Youden’s index
IDF	ATPⅢ	79.40	99.98	0.79	69.36	100	0.69	85.94	99.96	0.86
CDS	ATPⅢ	38.86	98.86	0.38	48.48	98.28	0.47	33.55	99.30	0.33

The highest consistency rate was between the IDF and ATPⅢ criteria (94.21%), followed by the IDF and CDS criteria (84.51%) and the ATPⅢ and CDS criteria (82.04%), and the kappa values was 0.85, 0.46 and 0.46, respectively. Overall, the kappa values indicated moderate or high consistencies (Table 5).

**Table 5:** Consistencies in the diagnoses of MS according to the three criteria.

Indexes	Total		Male		Female	
	N	Consistency rate (%)	N	Consistency rate (%)	N	Consistency rate (%)
IDF(+)ATPⅢ (+) / IDF(-)ATPⅢ (-)	5614	94.21	2269	92.57	3345	95.35
IDF(+)ATPⅢ (-)	1		0		1	
IDF(-)ATPⅢ(+)	344		182		162	
Kappa value		0.85*		0.77*		0.89*
IDF(+) CDS(+) / IDF(-) CDS(-)	5036	84.51	2169	88.49	2867	81.73
IDF(+) CDS(-)	776		187		589	
IDF(-) CDS(+)	147		95		52	
Kappa value		0.46*		0.55*		0.42*
ATPⅢ (+)CDS(+) / ATPⅢ (-) CDS(-)	4889	82.04	2113	86.21	2776	79.13
ATPⅢ (+) CDS(-)	1021		306		715	
ATPⅢ (-) CDS(+)	49		32		17	
Kappa value		0.46*		0.56*		0.40*

*P<0.05

### 3.4 The clusters of the MS risk factors prediction by applying WC and BMI cut-off points

MS risk factors clusters which was conformed to at least three conditions of ATPⅢ criterion was defined as State variables, and the BMI which indicated overweight or obesity in the CDS criterion and the WC which indicated central obesity in the IDF criterion was defined as Test Variable. We predict the MS risk factors clusters situation by continuous variables BMI and WC. The part of the analysis results at the Table 6 showed that it was more superior to predict the MS risk factors clusters by using BMI≥25kg/m^2^ indicator (sensitivity: 69.4%, specificity: 81.9%, distance of ROC: 0.36) in male than using WC≥90cm indicator (sensitivity: 62.3%, specificity: 90.8%, distance of ROC: 0.39). However, for female, it was superior by using WC≥80cm indicator (sensitivity: 80.9%, specificity: 72.5%, distance of ROC: 0.33) than using BMI≥25kg/m^2^ indicator (sensitivity: 52.3%, specificity: 84.4%, distance of ROC: 0.50). In fact, considering Jiangxi province residents (male/female), the ability of predicting MS risk factors clusters was superior when the cut-off point was 24/24kg/m^2^ using BMI indicator, otherwise when the cut-off point was 87/80cm using WC indicator. This was difference from the cut-off point (25 kg/m^2^) of BMI indicator in the CDS criterion and cut-off point (90/80 cm) of WC indicator in the IDF/ ATPⅢ criteria.

**Table 6:** Clustering of risk factors for MS predicted by cut-off point of WC and BMI.

	Cut-off point	Male			Female		
		Sensitivity (%)	Specificity (%)	Distance of ROC	Sensitivity (%)	Specificity (%)	Distance of ROC
BMI	20kg/m^2^	99.0	21.3	0.79	97.1	20.2	0.80
	21kg/m^2^	96.8	33.8	0.66	94.5	33.4	0.67
	22kg/m^2^	93.3	48.3	0.52	88.5	48.6	0.53
	23kg/m^2^	88.6	61.2	0.40	78.6	62.7	0.43
	^#^24kg/m^2^	81.8	72.7	0.33	66.1	74.9	0.42
	25kg/m^2^	69.4	81.9	0.36	52.3	84.4	0.50
	26kg/m^2^	55.2	89.1	0.46	38.5	89.6	0.62
	27kg/m^2^	39.7	93.1	0.61	25.7	93.2	0.75
	28kg/m^2^	24.4	96.8	0.76	17.5	95.6	0.83
WC	77cm	96.3	38.1	0.62	90.9	56.0	0.45
	78cm	95.1	43.2	0.57	88.4	61.6	0.40
	79cm	93.9	48.5	0.52	86.5	67.4	0.35
	**80cm	92.8	53.7	0.47	80.9	72.5	0.33
	81cm	90.7	58.0	0.43	75.1	76.1	0.34
	82cm	88.9	62.7	0.39	69.7	79.3	0.37
	83cm	87.2	67.3	0.35	63.5	82.5	0.40
	84cm	85.0	71.4	0.32	58.4	84.8	0.44
	85cm	82.5	75.3	0.30	51.7	87.2	0.50
	86cm	80.0	79.5	0.29	45.7	89.2	0.55
	*87cm	77.4	82.7	0.28	41.3	91.2	0.59
	88cm	74.4	85.7	0.29	36.6	92.5	0.64
	89cm	70.9	88.3	0.31	31.4	93.7	0.69
	90cm	62.3	90.8	0.39	27.2	94.8	0.73
	91cm	55.4	92.4	0.45	24.3	95.7	0.76
	92cm	48.1	94.0	0.52	20.5	96.8	0.80

At least three of the risk factors aggregation refer to ATPⅢ(2005) criterion.

^#^ The optimal cut-off point of BMI for male and female.

* The optimal cut-off point of WC for male.

** The optimal cut-off point of WC for female.

All the individuals were divided by age and sex, and those who met at least three of the ATPⅢ criterion were regarded as the clusters of risk factors for MS; The BMI (25 kg/m^2^) of the CDS criterion for determining overweight or obesity were selected, and those of the IDF criteria for central obesity in male/ female WC (90 / 80cm) were adopted, and prediction ability of MS risk factors clusters between them were compared. The results showed that BMI and WC had different ability to predict clusters of risk factors for MS in different age groups. Among the 18~59 years old male group, BMI was superior to WC in predicting clusters of risk factors for MS; while in the 60 years and above male group and all-age female group, WC was superior to BMI. (Table 7)

**Table 7:** The clusters of risk factors for MS predicted by cut-off point of WC and BMI in different age groups.

	Age group	Male			Female		
		Sensitivity (%)	Specificity (%)	Distance of ROC	Sensitivity (%)	Specificity (%)	Distance of ROC
BMI≥25kg/m^2^	18~44	78.8	78.5	0.30	65.0	85.7	0.38
	45~59	66.4	79.4	0.39	54.7	81.9	0.49
	60~	61.8	88.9	0.40	40.8	87.1	0.61
WC≥90/80cm	18~44	68.5	89.3	0.33	86.5	76.5	0.27
	45~59	58.1	89.6	0.43	79.9	69.3	0.37
	60~	61.8	94.0	0.39	79.2	70.6	0.36

At least three of the risk factors clusters refer to ATPⅢ(2005) criterion.

## 4 Discussion

The prevalence rate of MS standardized using the IDF, ATPⅢ and CDS criteria for the diagnoses of the adult population was 19.85%, 24.77% and 9.95%, respectively, in Jiangxi Province, and these values were close to the reported prevalence rates of MS in Guangdong Province[13] (20.3%, 24.3%, and 11.4%, respectively), lower than those in the Jiangsu Province[14] (24.6%, 31.0%, and 15.1%, respectively) and higher than those in the 6 counties of Yunnan province[15](13.1%, 16.6% and 10.0%, respectively) with the exception of the CDS criterion. Compared to research results from abroad, the rates were lower than those of Turkish [16] (44.0% and 36.6% according to the IDF and ATPⅢ criteria, respectively), Australian[17], Malaysian[18] and Jordanian adults[19] (30.7%, 37.4%, and 51%, respectively, according to the IDF criterion), and higher than those of the urban adult population of Brazil[20] (22.7% according to the ATPⅢ criterion). The prevalence rates of MS among female were higher than that of male according to both the IDF and ATPⅢ criteria, which contrasted with the result based on the CDS criterion, which was consistent with Li’s [21] research. The most prevalent component of MS was elevated blood pressure according to the IDF and ATPⅢ criteria, while the prevalent of overweight or obesity was the most prevalent component according to the CDS criterion. Other research conducted in Guangdong and Jiangsu[22] has come to the same conclusions. We believe that the large number of high-risk patients with hypertension should prompt the focus of more attention to the prevention and control of blood pressure. Anecdotal evidence[23]indicates that increased sodium intake is a risk factor for hypertension. With rapid economic development, the accelerating pace of life, irregular diets and a sedentary lifestyle, a large number of the overweight or obese population has health problems that cannot be ignored as detected by the CDS criterion.

At present, the war regarding the distinct criteria for the diagnosis of MS continues. However, all of this war is based on the cut-off points and focuses. Central obesity is a prerequisite for the diagnosis according to the IDF criterion, whereas WC is used as a measure of adiposity. The ATPⅢ criterion include the same cut-off points for WC as the IDF criterion, but central obesity is eliminated as a prerequisite. MS pathogenesis according to the ATPⅢ criterion extends to more cardiovascular risk factors than central obesity alone[24]. Additionally, the ATPⅢ criterion include people who have received treatment for hyperglycemia. The other component cut-off points are consistent between the ATPⅢ and IDF criteria. BMI is used to diagnose overweight or obesity in the CDS criteria. The blood pressure and blood glucose cut-off points in the CDS criteria are both higher than those of the other two criteria, while the HDL-C cut-off points for male and female are lower in the CDS criterion than the other two criteria. In addition, the content of the CDS criterion added the cut-off point of 2-hour postprandial plasma glucose. The prevalence rate of MS and its components were different according to the three criteria. The highest prevalence rate of MS was found using the ATPⅢ criterion followed by the IDF criterion and the CDS criterion. These results are similar to those of other studies[25,26] that used the same criteria. Additionally, The results indicated the IDF criteria was more superior to distinguish the true MS patients and non-patients than CDS criterion when referring the ATPⅢ criterion, and the consistency rate between IDF and ATPⅢ criteria was the highest. It may be related to the fact that the IDF and ATPⅢ criteria for most components were the same. The kappa values also exhibited moderate and even high consistencies among the three sets of criteria.

One of the greatest controversy among these three criteria was whether the WC or BMI should be used as the measurement of obesity. The WC is one of the simplest indicators and reflects visceral abdominal fat accumulation. However, the WC index does not include a measurement of height and thus may have limitations in predicting cardiovascular risk factors for the tall and short populations[27]. BMI has been used as an indicator in chronic disease surveillance and nutrition surveys in China[28] and has its own distinct advantages, such as its measurement is easy to master, and it is more suitable for use in primary medical institutions. But the BMI was unable to differentiate between lean mass and fat mass[29].

The results of this study showed that BMI and WC predict the clusters of risk factors for MS in different gender. The best cut points are 24/24 kg / m^2^(male / female) and 87 / 80cm (male / female), respectively. The ROC curve distance (0.28 / 0.33) for WC was shorter than the ROC curve distance for BMI (0.33 / 0.42). Our study showed that WC is superior to BMI in the identification of MS. The same results were also shown in some cross-sectional surveys [30, 31]. However, if the CDS criterion in BMI (more than 25kg/m^2^) and IDF in male / female WC (more than 90/80cm) were compared, the ROC curve of the distance suggested that male who using BMI (more than 25 kg/m^2^) were superior to WC (more than 90cm), while the female who using WC (more than 90cm) were better than BMI (25 = kg/m^2^). The results of this study showed that using IDF and CDS criteria to determine the obesity (take ATPⅢ criterion as a reference), the male population who using the CDS criterion is more conducive to identify MS patients, while IDF criterion is more suitable for identify MS in female.

This study also found that BMI and WC had different ability to predict clusters of risk factors for MS in different age groups. In the 18~59 years old male group, BMI was superior to WC in predicting MS risk factors clusters; while in the 60 years and above male group and all-age female group, WC was superior to BMI. The reason may be: male who aged 18~59 years old was the main labor crowd, with more individual labor volume or physical exercise, thus, they remained relatively lower fat content than other groups. The predictive power of BMI for obesity was superior than that of WC. When the male arrived at 60~ years old, the lifestyle changed, the amount of labor decreased, the individual entered the aging stage, with subcutaneous fat decreased, visceral abdominal fat ratio increased, and the WC prediction ability of MS was superior than that of BMI. On the one hand, the body fat percent in female population was higher than that in adult male [32]. On the other hand, with the increasing of age, especially those female who experienced menopause period, the distribution of fat changes, and more abdominal fat were aggregated [33]. Therefore, the ability of WC index to predict clusters of risk factors for MS among all female age groups was superior than that of BMI. However, in the diagnosis of MS, whether different indicators and cut-off point should be used for different age and gender groups separately to diagnose obesity still needs to be further studied.

Among the three criteria for MS, there was bigger difference in applicability for the adults of Jiangxi Province. However, the IDF criterion may focus more on clinical practice, the ATPⅢ criterion focuses on prevention in the population, and the CDS criterion emphasizes the characteristics of the Chinese people[24]. The choice of the appropriate criteria should be based on the actual conditions of the site. The practical values of the three criteria should be evaluated by many prospective and follow-up studies to further confirm this notion. Additionally, our results revealed the high prevalence rate of metabolic syndrome among adults in Jiangxi Province, which emphasizes the great importance of prevention and control. Especially for the high-risk groups with high blood pressure and obesity, it is necessary to strengthen health education, advocate balanced diets, decrease salt intake, and encourage people to actively participate in nationwide fitness campaigns with the goal of losing weight.

An important limitation of the present study is the cross-sectional design. This fact limited our ability to draw any causal conclusions. Moreover, this study did not adopt new criteria such as JCDCG [34] and JIS [35], which may lead to the underestimation of the prevalence of MS among adult inhabitants in Jiangxi province. But ourr study individuals were selected based on the geographic, disease distribution, and demographic characteristics, which guaranteed the sample representative to some extent. The sample size is also large. At the same time, the investigators received unified training, the investigation process was strictly controlled by quality, and the data results were authentic and reliable.

## Supporting information

S1 Table The three criteria for MS. (DOCX)

S2 Table Characteristics of the participants. (DOCX)

S3 Table The prevalent rates of the components of MS by gender (according to the IDF, ATⅢ, and CDS criteria). (DOCX)

S4 Table Analysis on validity of different criteria for MS. (DOCX)

S5 Table Consistencies in the diagnoses of MS according to the three criteria. (DOCX)

S6 Table Clustering of risk factors for MS predicted by cut-off point of WC and BMI. (DOCX)

S7 Table The clusters of risk factors for MS predicted by cut-off point of WC and BMI in different age groups. (DOCX)

S8 Table Questionnaire1 (original language). (PDF)

S9 Table Questionnaire 2 (English). (DOCX)
