# Supplementary material for: Comparative analysis of IDF, ATPIII and CDS in the diagnosis of metabolic syndrome among adult inhabitants in Jiangxi Province, China
Source: PLoS One. 2017 Dec 7;12(12):e0189046. doi: 10.1371/journal.pone.0189046 (PMC5720703; doi:10.1371/journal.pone.0189046)
Supplement: S1 Table — (DOCX) [file pone.0189046.s001.docx]

**Table 1. The three criteria for MS.**

| Components | IDF (2005) | ATPⅢ(2005) | CDS (2004) |
| --- | --- | --- | --- |
| Obesity (Chinese) | waist circumference (WC)≥90cm (male) or ≥80 cm (female) | 1.waist circumference (WC)≥90 cm (male) or ≥80 cm (female) | 1.BMI≥25.0 kg/m^2^ |
| Elevated blood pressure | 1.systolic blood pressure (SBP)≥130 or diastolic blood pressure (DBP) ≥85 mmHg or treatment of previously diagnosed hypertension | 2.systolic blood pressure (SBP)≥130 or diastolic blood pressure (DBP)≥85 mmHg or on antihypertensive drug treatment in a patient with a history of hypertension | 2.systolic blood pressure (SBP)≥140ordiastolic blood pressure (DBP)≥90mmHg and (or) treatment of previously diagnosed hypertension |
| Hyperglycemia | 2.fasting plasma glucose (FPG) ≥ 100 mg/dL (5.6) mmol/L), or previously diagnosed type 2 diabetes | 3.fasting plasma glucose (FPG)≥100 mg/dL (5.6mmol/L) or on drug treatment for elevated glucose | 3.fasting plasma glucose (FPG)≥110 mg/dL (6.1 mmol/L) and (or) 2 hours postprandial plasma glucose (2hPPG)≥140 mg/dL (7.8 mmol/L), and (or) on drug treatment for diabetes |
| Dyslipidemia | 3.triglyceride (TG)≥ 150 mg/dL (1.7 mmol/L) orspecific treatment for this lipid abnormality at high-density4.high density lipoprotein cholesterol (HDL-C)< 40 mg/dL (1.03 mmol/L) (male),<50 mg/dL (1.29 mmol/L)(female)or specific treatment for this lipid abnormality | 4.triglyceride (TG)≥ 150 mg/dL (1.7 mmol/L) or on drug treatment for elevated triglycerides 5.high density lipoprotein cholesterol (HDL-C)< 40 mg/dL (1.03 mmol/L) (male),<50 mg/dL (1.3 mmol/L) (female)or on drug treatment for reduced HDL | 4.triglyceride(TG)≥ 150 mg/dL (1.7 mmol/L) and (or) high density lipoprotein cholesterol (HDL-C)< 35 mg/dL (0.9 mmol/L) (male)< 39 mg/dL (1.0 mmol/L) (female) |

Note: MS was diagnosed as follows: the IDF criterion: a person with abdominal obesity (waist circumference≥90 cm (male) or ≥80 cm (female), plus any two risk factors labeled 1~4; the ATPⅢ criterion: a person with at least three of the factors labeled 1~5; and the CDS criterion: a person with at least three of the factors labeled 1~4.
