# Supplementary material for: Comparative analysis of IDF, ATPIII and CDS in the diagnosis of metabolic syndrome among adult inhabitants in Jiangxi Province, China
Source: PLoS One. 2017 Dec 7;12(12):e0189046. doi: 10.1371/journal.pone.0189046 (PMC5720703; doi:10.1371/journal.pone.0189046)
Supplement: S2 Table — (DOCX) [file pone.0189046.s002.docx]

**Table 2. Characteristics of the participants.**

| Variables | Total | Male | Female | *P* |
| --- | --- | --- | --- | --- |
| N | 5959 | 2451 | 3508 | -- |
| Age (years) | 50.52±13.92 | 51.23±14.47 | 50.03±13.50 | 0.01^*^ |
| Height(cm) | 157.42±8.12 | 163.44±6.82 | 153.22±6.04 | <0.01^*^ |
| Weight(kg) | 57.95±10.48 | 62.56±10.89 | 54.74±8.86 | <0.01^*^ |
| WC (cm) | 80.80±9.33 | 82.73±9.52 | 79.46±8.96 | <0.01^*^ |
| BMI (kg/m^2^) | 23.31±3.32 | 23.34±3.33 | 23.28±3.31 | 0.48^*^ |
| SBP (mmHg) | 127.42±20.09 | 129.65±18.78 | 125.87±20.81 | <0.01^*^ |
| DBP (mmHg) | 75.42±11.12 | 77.61±11.43 | 73.88±10.63 | <0.01^*^ |
| FPG (mmol/L) | 5.52±1.33 | 5.56±1.32 | 5.49±1.34 | 0.05^*^ |
| 2hPPG (mmol/L) | 6.22±2.35 | 6.18±2.53 | 6.25±2.22 | 0.32^*^ |
| HDL-C (mmol/L) | 1.37±0.38 | 1.33±0.40 | 1.40±0.36 | <0.01^*^ |
| LDL-C(mmol/L) | 2.86±0.83 | 2.85±0.82 | 2.87±0.83 | 0.33^*^ |
| CHOL(mmol/L) | 4.66±0.93 | 4.65±0.92 | 4.67±0.94 | 0.42^*^ |
| TG (mmol/L) | 1.40±1.16 | 1.49±1.36 | 1.33±0.99 | <0.01^*^ |

^*^ Compared between male and female

The data are presented as the means ± the SDs.

WC, waist circumference; BMI, body mass index; SBP, systolic blood pressure; DBP, diastolic blood pressure; FPG, fasting plasma glucose; 2hPPG, 2-hour postprandial plasma glucose; HDL-C, high-density lipoprotein cholesterol; LDL-C, low-density lipoprotein cholesterol; CHOL, cholesterol; TG, triglycerides.
