# Supplementary material for: Comparative analysis of IDF, ATPIII and CDS in the diagnosis of metabolic syndrome among adult inhabitants in Jiangxi Province, China
Source: PLoS One. 2017 Dec 7;12(12):e0189046. doi: 10.1371/journal.pone.0189046 (PMC5720703; doi:10.1371/journal.pone.0189046)
Supplement: S3 Table — (DOCX) [file pone.0189046.s003.docx]

**Table 3. The prevalent rates of the components of MS by gender (according to the IDF, ATPⅢ, and CDS criteria).**

|  | Diagnostic criteria | Total  (5959) | | Male  (2451) | | Female  (3508) | | χ^2^ | *P* |
| --- | --- | --- | --- | --- | --- | --- | --- | --- | --- |
|  |  | Prevalence | Prevalence rate | Prevalence | Prevalence  rate | Prevalence | Prevalence  rate |  |  |
| Overweight or obese | CDS | 1685 | 28.28% | 747 | 30.48% | 938 | 26.74% | 9.89 | <0.01^*^ |
| Central obesity | IDF/ ATPⅢ | 2249 | 37.74% | 609 | 24.85% | 1640 | 46.75% | 294.01 | <0.01^*^ |
| Hypertriglyceridemia | IDF/ ATPⅢ | 1432 | 24.03% | 655 | 26.72% | 777 | 22.15% | 16.48 | <0.01^*^ |
| Reduced HDL-C | IDF | 2071 | 34.75% | 618 | 25.21% | 1453 | 41.42% | 167.62 | <0.01^*^ |
|  | ATPⅢ | 2117 | 35.53% | 618 | 25.21% | 1499 | 42.73% | 193.88 | <0.01^*^ |
| Elevated blood pressure | IDF/ ATPⅢ | 2483 | 41.67% | 1154 | 47.08% | 1329 | 37.88% | 51.26 | <0.01^*^ |
|  | CDS | 1656 | 27.79% | 744 | 30.35% | 912 | 26.00% | 14.01 | <0.01^*^ |
| Hyperglycemia | IDF | 1918 | 32.19% | 834 | 34.03% | 1084 | 30.90% | 6.31 | 0.01^*^ |
|  | ATPⅢ | 1912 | 32.09% | 833 | 33.99% | 1079 | 30.76% | 6.74 | 0.01^*^ |
|  | CDS | 1346 | 22.59% | 591 | 24.11% | 755 | 21.52% | 5.73 | 0.02^*^ |
| Dyslipidemia | CDS | 1524 | 25.57% | 670 | 27.34% | 854 | 24.34% | 6.74 | 0.01^*^ |
| MS | IDF | 1327 | 22.27% | 412 | 16.81% | 915 | 26.08% | 71.69 | <0.01^*^ |
|  | ATPⅢ | 1670 | 28.02% | 594 | 24.24% | 1076 | 30.67% | 29.65 | <0.01^*^ |
|  | CDS | 698 | 11.71% | 320 | 13.06% | 378 | 10.78% | 7.26 | 0.01^*^ |
| MS^**^ | IDF | -- | 19.85% | -- | 16.98% | -- | 22.11% | -- | -- |
|  | ATPⅢ | -- | 24.77% | -- | 23.86% | -- | 25.76% | -- | -- |
|  | CDS | -- | 9.95% | -- | 12.31% | -- | 8.50% | -- | -- |

^*^ Compared between the male and female.

^**^ Standardized prevalence rate of MS.
