# Supplementary material for: Comparative analysis of IDF, ATPIII and CDS in the diagnosis of metabolic syndrome among adult inhabitants in Jiangxi Province, China
Source: PLoS One. 2017 Dec 7;12(12):e0189046. doi: 10.1371/journal.pone.0189046 (PMC5720703; doi:10.1371/journal.pone.0189046)
Supplement: S4 Table — (DOCX) [file pone.0189046.s004.docx]

**Table 4 .Analysis on validity of different criteria for MS.**

| Test criteria | Reference criteria | Total | | | Male | | | Female | | |
| --- | --- | --- | --- | --- | --- | --- | --- | --- | --- | --- |
|  |  | Sensitivity (%) | Specificity (%) | Youden’s index | Sensitivity (%) | Specificity (%) | Youden’s index | Sensitivity (%) | Specificity (%) | Youden’s index |
| IDF | ATPⅢ | 79.40 | 99.98 | 0.79 | 69.36 | 100 | 0.69 | 85.94 | 99.96 | 0.86 |
| CDS | ATPⅢ | 38.86 | 98.86 | 0.38 | 48.48 | 98.28 | 0.47 | 33.55 | 99.30 | 0.33 |
