# Supplementary material for: Comparative analysis of IDF, ATPIII and CDS in the diagnosis of metabolic syndrome among adult inhabitants in Jiangxi Province, China
Source: PLoS One. 2017 Dec 7;12(12):e0189046. doi: 10.1371/journal.pone.0189046 (PMC5720703; doi:10.1371/journal.pone.0189046)
Supplement: S5 Table — (DOCX) [file pone.0189046.s005.docx]

**Table 5. Consistencies in the diagnoses of MS according to the three criteria.**

| Indexes | Total | | Male | | Female | |
| --- | --- | --- | --- | --- | --- | --- |
|  | N | Consistency rate (%) | N | Consistency rate (%) | N | Consistency rate (%) |
| IDF(+)ATPⅢ (+) / IDF(-)ATPⅢ (-) | 5614 | 94.21 | 2269 | 92.57 | 3345 | 95.35 |
| IDF(+)ATPⅢ (-) | 1 |  | 0 |  | 1 |  |
| IDF(-)ATPⅢ(+) | 344 |  | 182 |  | 162 |  |
| Kappa value |  | 0.85^*^ |  | 0.77^*^ |  | 0.89^*^ |
| IDF(+) CDS(+) / IDF(-) CDS(-) | 5036 | 84.51 | 2169 | 88.49 | 2867 | 81.73 |
| IDF(+) CDS(-) | 776 |  | 187 |  | 589 |  |
| IDF(-) CDS(+) | 147 |  | 95 |  | 52 |  |
| Kappa value |  | 0.46* |  | 0.55^*^ |  | 0.42^*^ |
| ATPⅢ (+)CDS(+) / ATPⅢ (-) CDS(-) | 4889 | 82.04 | 2113 | 86.21 | 2776 | 79.13 |
| ATPⅢ (+) CDS(-) | 1021 |  | 306 |  | 715 |  |
| ATPⅢ (-) CDS(+) | 49 |  | 32 |  | 17 |  |
| Kappa value |  | 0.46^*^ |  | 0.56^*^ |  | 0.40^*^ |

^*^*P*<0.05
