# Supplementary material for: Comparative analysis of IDF, ATPIII and CDS in the diagnosis of metabolic syndrome among adult inhabitants in Jiangxi Province, China
Source: PLoS One. 2017 Dec 7;12(12):e0189046. doi: 10.1371/journal.pone.0189046 (PMC5720703; doi:10.1371/journal.pone.0189046)
Supplement: S6 Table — (DOCX) [file pone.0189046.s006.docx]

**Table 6.Clustering of risk factors for MS predicted by cut-off point of WC and BMI.**

|  | Cut-off point | Male | | | Female | | |
| --- | --- | --- | --- | --- | --- | --- | --- |
|  |  | Sensitivity (%) | Specificity (%) | Distance of ROC | Sensitivity (%) | Specificity (%) | Distance of ROC |
| BMI | 20kg/m^2^ | 99.0 | 21.3 | 0.79 | 97.1 | 20.2 | 0.80 |
|  | 21kg/m^2^ | 96.8 | 33.8 | 0.66 | 94.5 | 33.4 | 0.67 |
|  | 22kg/m^2^ | 93.3 | 48.3 | 0.52 | 88.5 | 48.6 | 0.53 |
|  | 23kg/m^2^ | 88.6 | 61.2 | 0.40 | 78.6 | 62.7 | 0.43 |
|  | ^#^24kg/m^2^ | 81.8 | 72.7 | 0.33 | 66.1 | 74.9 | 0.42 |
|  | 25kg/m^2^ | 69.4 | 81.9 | 0.36 | 52.3 | 84.4 | 0.50 |
|  | 26kg/m^2^ | 55.2 | 89.1 | 0.46 | 38.5 | 89.6 | 0.62 |
|  | 27kg/m^2^ | 39.7 | 93.1 | 0.61 | 25.7 | 93.2 | 0.75 |
|  | 28kg/m^2^ | 24.4 | 96.8 | 0.76 | 17.5 | 95.6 | 0.83 |
| WC | 77cm | 96.3 | 38.1 | 0.62 | 90.9 | 56.0 | 0.45 |
|  | 78cm | 95.1 | 43.2 | 0.57 | 88.4 | 61.6 | 0.40 |
|  | 79cm | 93.9 | 48.5 | 0.52 | 86.5 | 67.4 | 0.35 |
|  | ^**^80cm | 92.8 | 53.7 | 0.47 | 80.9 | 72.5 | 0.33 |
|  | 81cm | 90.7 | 58.0 | 0.43 | 75.1 | 76.1 | 0.34 |
|  | 82cm | 88.9 | 62.7 | 0.39 | 69.7 | 79.3 | 0.37 |
|  | 83cm | 87.2 | 67.3 | 0.35 | 63.5 | 82.5 | 0.40 |
|  | 84cm | 85.0 | 71.4 | 0.32 | 58.4 | 84.8 | 0.44 |
|  | 85cm | 82.5 | 75.3 | 0.30 | 51.7 | 87.2 | 0.50 |
|  | 86cm | 80.0 | 79.5 | 0.29 | 45.7 | 89.2 | 0.55 |
|  | ^*^87cm | 77.4 | 82.7 | 0.28 | 41.3 | 91.2 | 0.59 |
|  | 88cm | 74.4 | 85.7 | 0.29 | 36.6 | 92.5 | 0.64 |
|  | 89cm | 70.9 | 88.3 | 0.31 | 31.4 | 93.7 | 0.69 |
|  | 90cm | 62.3 | 90.8 | 0.39 | 27.2 | 94.8 | 0.73 |
|  | 91cm | 55.4 | 92.4 | 0.45 | 24.3 | 95.7 | 0.76 |
|  | 92cm | 48.1 | 94.0 | 0.52 | 20.5 | 96.8 | 0.80 |

At least three of the risk factors aggregation refer to ATPⅢ(2005) criterion.

^#^ The optimal cut-off point of BMI for male and female.

^*^ The optimal cut-off point of WC for male.

^**^ The optimal cut-off point of WC for female.
