# Supplementary material for: Comparative analysis of IDF, ATPIII and CDS in the diagnosis of metabolic syndrome among adult inhabitants in Jiangxi Province, China
Source: PLoS One. 2017 Dec 7;12(12):e0189046. doi: 10.1371/journal.pone.0189046 (PMC5720703; doi:10.1371/journal.pone.0189046)
Supplement: S7 Table — (DOCX) [file pone.0189046.s007.docx]

**Table 7. The clusters of risk factors for MS predicted by cut-off point of WC and BMI in different age groups.**

|  | Age group | Male | | | Female | | |
| --- | --- | --- | --- | --- | --- | --- | --- |
|  |  | Sensitivity (%) | Specificity (%) | Distance of ROC | Sensitivity (%) | Specificity (%) | Distance of ROC |
| BMI≥25kg/m^2^ | 18~44 | 78.8 | 78.5 | 0.30 | 65.0 | 85.7 | 0.38 |
|  | 45~59 | 66.4 | 79.4 | 0.39 | 54.7 | 81.9 | 0.49 |
|  | 60~ | 61.8 | 88.9 | 0.40 | 40.8 | 87.1 | 0.61 |
| WC≥90/80cm | 18~44 | 68.5 | 89.3 | 0.33 | 86.5 | 76.5 | 0.27 |
|  | 45~59 | 58.1 | 89.6 | 0.43 | 79.9 | 69.3 | 0.37 |
|  | 60~ | 61.8 | 94.0 | 0.39 | 79.2 | 70.6 | 0.36 |

At least three of the risk factors clusters refer to ATPⅢ(2005) criterion.
