# Supplementary material for: Comparative analysis of IDF, ATPIII and CDS in the diagnosis of metabolic syndrome among adult inhabitants in Jiangxi Province, China
Source: PLoS One. 2017 Dec 7;12(12):e0189046. doi: 10.1371/journal.pone.0189046 (PMC5720703; doi:10.1371/journal.pone.0189046)
Supplement: S8 Table — (PDF) [file pone.0189046.s008.pdf]

是否糖尿病：1 是 2 否

贴问卷编码条处

根据中华人民共和国《统计法》第三章第十五条规定，“属于私人、家庭的单项调查资料，非经本人同意，不得外泄”。

中国慢性病及其危险因素监测（2013）

个人问卷

调查对象姓名：__________	电话：__________
监测点名称（县/区）：__________	监测点代码：□□□□□□
乡镇/街道名称：__________	乡镇/街道代码：□
村/居委会名称：__________	村/居委会代码：□
	家庭代码：□□
调查员签名：__________	日期：2013 年□□月□□日
监测点质控员签名：__________	日期：2013 年□□月□□日
省级督导员签名：__________	日期：2013 年□□月□□日

中国疾病预防控制中心
慢性非传染性疾病预防控制中心
二〇一三年六月

贴采血编码条处

调查开始时间 （24 小时制）：□□ 时 □□ 分

**第一部分　基本信息**		
A1	您的出生日期<br>调查员注意：哪项记不清则在相应项内靠右填“-9”	□□□□ 年 □□ 月 □□ 日
A2	性别	1　男<br>2　女
A3	您的民族	1　汉族<br>2　壮族<br>3　满族<br>4　回族<br>5　苗族<br>6　维吾尔族<br>7　彝族<br>8　土家族<br>9　蒙古族<br>10　朝鲜族<br>11　藏族<br>88　其他民族
A4	您的文化程度	1　未接受正规学校教育<br>2　小学未毕业<br>3　小学毕业<br>4　初中毕业<br>5　高中/中专/技校<br>6　大专毕业<br>7　本科毕业<br>8　研究生及以上
A5	您目前的婚姻状况	1　未婚<br>2　已婚<br>3　同居<br>4　丧偶<br>5　离婚<br>6　分居
A6	您的职业	1　农林牧渔水利业生产人员<br>2　生产、运输设备操作人员及有关人员<br>3　商业、服务业人员<br>4　国家机关、党群组织、企业、事业单位负责人<br>5　办事人员和有关人员<br>6　专业技术人员<br>7　军人<br>8　其他劳动者<br>9　在校学生<br>10　未就业<br>11　家务<br>12　离退休人员
A7	您目前参加了哪种医疗保险?（可多选）<br>调查员注意：须读出答案	1　城镇职工基本医疗保险<br>2　公费医疗<br>3　城镇居民医疗保险<br>4　新型农村合作医疗<br>5　商业医疗保险<br>6　其他<br>7　没参加<br>99　不清楚
A8	您的户籍所在地是？	1　本县（区）<br>2　在本地市所属其他区<br>3　在本地市所属其他县<br>4　在本省（自治区、直辖市）所属其他地市<br>5　在其他省（自治区、直辖市）

第二部分　吸烟情况

**现在吸烟情况**			
B1	您现在吸烟吗，每天吸、不是每天吸、还是不吸？	1　是的，每天吸<br>2　是的，但不是每天吸……..………➔<br>3　以前吸，但现在不吸………..……➔<br>4　从不吸　　……………………..……➔	 B3<br>B8<br>B11
B2	您是从什么时候开始每天吸烟的？<br>**调查员注意：“记不清”填“-9 ”**	□□周岁	
B3	您现在平均每天（每周）吸多少支机制卷烟？<br>**调查员注意：每日吸烟者回答选项 1，非每日吸烟者回答选项 2**	1　□□支/天<br>2　□□支/周<br>3　不吸机制卷烟	
**戒烟行为**			
B4	过去您是否戒过烟？（这里的戒烟指认真考虑过要戒烟并有所行动）	1　是，过去 12 个月内<br>2　是，12 个月以前………..…………➔<br>3　否　………….…………..…………➔	 B6<br>B6
B5	过去 12 个月内，您是否使用过尼古丁替代治疗或其他西药尝试戒烟？	1　是<br>2　否	
B6	下面哪个选项最符合您关于戒烟的想法？	1　准备在一个月内戒烟<br>2　考虑在 12 个月内戒烟<br>3　会戒烟，但不会在 12 个月内<br>4　不想戒烟<br>99　不知道	
B7	在过去的 12 个月内，您看病时，医护人员是否建议您戒烟？	1　没有看过病…………………………➔<br>2　看病时医护人员曾建议戒烟………➔<br>3　看病时医护人员没有建议戒烟……➔	B11<br>B11<br>B11
B8	您停止吸烟多长时间了？<br>（**调查员注意：仅包括调查对象完全戒烟的情况，还在偶尔吸烟的情况不包括在内。注意只能填写一项**）	a □□年<br>b □□月<br>c □□周<br>d □□日	
	**调查员注意：如果 B8<1 年（<12 个月）**　......……....……………......…......……......…➔<br>**否则**　……...……......…......……......……………......…......…......……......…➔		B9<br>B11
B9	在过去的 12 个月内，您看病时，医护人员是否建议您戒烟？	1　没有看过病<br>2　看病时医生曾建议戒烟<br>3　看病时医生没有建议戒烟	
B10	在过去 12 个月内，您是否曾使用过尼古丁替代治疗或其他西药尝试戒烟？	1　是<br>2　否	

二手烟暴露		
B11	通常情况下，您每周接触二手烟的天数是？（二手烟是指吸烟时，吸烟者呼出的以及卷烟末端散发出的烟雾）	1 每天<br>2 平均每周有 4-6 天<br>3 平均每周有 1-3 天<br>4 没有<br>99 不知道/记不清

知识、态度和认识				
B12	据您所知，吸烟会不会造成严重的疾病？	1 会<br>2 不会 ……...………….………….➔<br>99 不知道 ……..………….………….➔		 B14<br>B14
B13 据您所知，吸烟会不会造成以下疾病？				
		会	不会	不知道/不确定
a	中风（脑卒中，脑血栓）	1	2	99
b	心脏病发作	1	2	99
c	肺癌	1	2	99
B14	据您所知，吸入二手烟烟雾会不会造成严重的疾病？	1 会<br>2 不会 ……...………….………….➔<br>99 不知道 ……..………….………….➔		 B16<br>B16
B15 据您所知，吸入二手烟烟雾会不会引起下列疾病？				
		会	不会	不知道/不确定
a	成人心脏疾病	1	2	99
b	儿童肺部疾病	1	2	99
c	成人肺癌	1	2	99
B16	您是否同意标明低焦油含量卷烟的危害比一般卷烟小？	1 同意<br>2 不同意<br>99 不知道		

第三部分 饮酒情况

C1	过去 12 个月里，您喝过酒吗？	1 喝过，在过去 30 天以前<br>2 喝过，在 30 天内<br>3 没喝过 ……………………….➔	 <br>D1
C2	过去 12 个月里，您饮酒的频率如何？<br>（调查员注意：须读出选项）	1 每天<br>2 5-6 天/周<br>3 3-4 天/周<br>4 1-2 天/周<br>5 1-3 天/月<br>6 少于 1 天/月	

请回答:过去 12 个月里，下列酒类您通常的饮用频率，通常一天喝多少？

调查员注意：记不清在小数点前靠右填“-9”，没有饮用则不填饮用频率和饮酒量

		a 是否饮用<br>1 是，2 否	b 饮用频率（**只填其中 1 项**）<br>b1<br>天/周	 b2<br>天/月	 b3<br>天/12 月	过去 12 个月中饮酒的日子里，通常一天的饮用量
C3	a. 白酒（≥42 度）	□	□	□□	□□□	□□.□两
	b. 白酒（<42 度）	□	□	□□	□□□	□□.□两
	c. 啤酒（580ml/瓶，4 度）	□	□	□□	□□□	□□.□瓶
	d. 黄酒（18 度）	□	□	□□	□□□	□□.□两
	e. 米酒（18 度）	□	□	□□	□□□	□□.□两
	f. 葡萄酒（10 度）	□	□	□□	□□□	□□.□两
	g. 青稞酒（3 度）	□	□	□□	□□□	□□.□两

C4	**a.对男性：**<br>过去 12 个月里，您一次喝酒超过 2.5 两高度白酒，或 3.5 两低度白酒，或 3 瓶啤酒，或 5 个易拉罐啤酒，或 7.5 两黄酒/米酒，或 1 斤半葡萄酒，或 3 斤青稞酒的频率如何？	1 每天或几乎每天（≥5 天/周）<br>2 1-4 天/周<br>3 1-3 天/月<br>4 低于 1 天/月<br>5 从未
	**b.对女性：**<br>过去 12 个月里，您一次喝酒超过 2 两高度白酒，或 3 两低度白酒，或 2.5 瓶啤酒，或 4 个易拉罐啤酒，或 6 两黄酒/米酒，或 1 斤 2 两葡萄酒，或 2.5 斤青稞酒的频率如何？	1 每天或几乎每天（≥5 天/周）<br>2 1-4 天/周<br>3 1-3 天/月<br>4 低于 1 天/月<br>5 从未

第四部分 饮食情况

D1	过去 12 个月里，您通常一天吃几顿饭？	□顿		
		就餐地点		
		a 家	b 食堂	c 餐馆
D2	过去 12 个月里，您通常一周在不同就餐地点吃早餐的天数？	□天	□天	□天
D3	过去 12 个月里，您通常一周在不同就餐地点吃午餐的天数？	□天	□天	□天
D4	过去 12 个月里，您通常一周在不同就餐地点吃晚餐的天数？	□天	□天	□天

请回忆在过去 12 个月里通常情况下，您是否吃过下列食物，并估计各类食物的食用频率和食用量。							
		a 是否食用<br>1 是，2 否	b 食用频率（**只填其中 1 项**）				平均每次食用量
			b1<br>次数/天	b2<br>次数/周	b3<br>次数/月	b4<br>次数/年	
D5	猪肉<br>（按生重记录）	□	□	□	□	□□	□□.□两
D6	牛、羊等畜肉<br>（按生重记录）	□	□	□	□	□□	□□.□两
D7	禽肉<br>（按生重记录）	□	□	□	□	□□	□□.□两
D8	水产品<br>（按生重记录）	□	□	□	□	□□	□□.□两
D9	新鲜蔬菜	□	□	□	□	□□	□□.□两
D10	新鲜水果	□	□	□	□	□□	□□.□两
D11	含糖碳酸饮料<br>（250ml/杯）	□	□	□	□	□□	□□.□杯
D12	果汁/果味饮料<br>（250ml/杯）	□	□	□	□	□□	□□.□杯

D13	您知道中国居民膳食指南推荐成人每人每天吃盐不应超过几克吗？	1 知道，为□□克<br>88 不知道
D14	您觉得多吃盐会加重或引起下列哪些疾病？<br>（**可多选**）	1 高血压<br>2 脑卒中<br>3 心肌梗死<br>4 肾脏病<br>5 都无关<br>88 其他<br>99 不清楚
D15	您认为自己吃盐过多吗？	1 较少<br>2 适中<br>3 过多<br>99 不清楚
D16	如果您知道多吃盐有害健康的话，您愿意少吃盐么？	1 愿意<br>2 不愿意<br>3 无所谓<br>99 不清楚
D17	您是否采取过减盐措施？如果是，您具体采取了哪些措施？<br>（**可多选**）	1 未采取任何减盐措施<br>2 减少外出吃饭<br>3 烹调食物时少放盐<br>4 少吃含盐高的食物，如腌制食品、豆腐乳、咸鸭蛋、大酱、黄酱等<br>5 在餐桌上吃饭时不再额外加任何盐<br>6 使用限盐工具，如控盐勺<br>7 使用低钠盐<br>88 其他

第五部分　身体活动

下列问题是通常一周您进行各类身体活动（包括干农活、工作、家务、交通相关的身体活动、休闲性锻炼或运动等）的情况。请回答：

工作、农业及家务性身体活动

E1	在您的工作、农活及家务活动中，有没有高强度活动，并且活动时间持续10分钟以上？<br>（高强度活动是指如搬运重物、挖掘等需要付出较大体力，或引起呼吸、心跳显著增加的活动）<br>**调查员注意：可出示身体活动分类表。**	1　有<br>2　没有　…………………...…➔	E4
E2	在您的工作、农活及家务活动中，通常一周内有多少天会进行上述高强度活动？	□ 天	
E3	在您的工作、农活及家务活动中，通常一天内累计有多长时间进行上述高强度活动？<br>**调查员注意：每次活动时间若少于 10 分钟，则不计算在内。**	□□ 小时 □□ 分钟	
E4	在您的工作、农活及家务活动中，有没有中等强度活动,并且活动时间持续10分钟以上？<br>（中等强度活动是指如锯木头、洗衣服、打扫卫生等需要付出中等体力，或引起呼吸、心跳轻度增加的活动）<br>**调查员注意：可出示身体活动分类表。**	1　有<br>2　没有　…………………...…➔	E7
E5	在您的工作、农活及家务活动中，通常一周内有多少天会进行上述中等强度活动？	□ 天	
E6	在您的工作、农活及家务活动中，通常一天内累计有多长时间进行上述中等强度活动？<br>**调查员注意：每次活动时间若少于 10 分钟，则不计算在内。**	□□ 小时 □□ 分钟	

交通性身体活动

以下问题不包括上述已提及的农业性身体活动和工作及家务性身体活动。

E7	您在外出时，有没有步行或骑自行车持续至少 10 分钟的情况？	1　有<br>2　没有　…………………...…➔	E10
E8	通常一周内，您有多少天外出时步行或骑自行车持续至少 10 分钟？	□ 天	

E9	通常一天内，您步行或骑自行车多长时间？	□□小时□□分钟	
**休闲性身体活动**<br>以下问题不包括上述已提及的农业性、工作、家务和交通性的身体活动。			
E10	您是否进行持续至少 10 分钟，引起呼吸、心跳显著增加的高强度活动吗？如长跑、游泳、踢足球等。<br>**调查员注意：可出示身体活动分类表。**	1 有<br>2 没有 ………………...…➔	E13
E11	通常一周内，您有多少天进行上述高强度的运动或休闲活动？	□天	
E12	通常一天内，您累计有多长时间进行上述高强度的运动或休闲活动？	□□小时□□分钟	
E13	您是否进行持续至少 10 分钟，引起呼吸、心跳轻度增加的中等强度运动和休闲活动吗？如快步走、打太极拳等。<br>**调查员注意：可出示身体活动分类表。**	1 有<br>2 没有 ………………...…➔	E16
E14	通常一周内，您有多少天进行上述中等强度的运动或休闲活动？	□天	
E15	通常一天内，您累计有多长时间进行上述中等强度的运动或休闲活动？<br>**调查员注意：每次活动时间若少于 10 分钟，则不计算在内。**	□□小时□□分钟	

**总静态行为**		
E16	通常一天内，您累计有多少时间坐着、靠着或躺着？（包括坐着工作、学习、阅读、看电视、用电脑、休息等所有静态行为的时间，但不包括睡觉时间）	□□小时□□分钟
**业余时间静态行为**		
E17a	您在业余时间里，平均每天看电视的时间为多少？	□□小时□□分钟
E17b	您在业余时间里，平均每天使用电脑（包括台式电脑、笔记本电脑、平板电脑等）的时间为多少？	□□小时□□分钟
E17c	您在业余时间里，平均每天使用手机的时间为多少？	□□小时□□分钟
E17d	您在业余时间里，平均每天用于阅读（纸质读物）的时间为多少？	□□小时□□分钟

睡眠行为		
E18	通常一天内，您睡觉累计有多少时间？	□□小时 □□分钟

第六部分　体重、血压、血糖、血脂等信息

F1 体重及其控制			
F1a	您最近一次测量体重的时间是？	1 从未量过<br>2 7 天内<br>3 1 个月内<br>4 3 个月内<br>5 6 个月内<br>6 12 个月以内<br>7 12 个月以前<br>99 记不清	
F1b	您的体重与 12 个月之前比有什么变化吗？	1 增加了 2.5 公斤或以上<br>2 基本保持不变（增减在 2.5 公斤以内）<br>3 下降了 2.5 公斤以上<br>99 不知道	
F1c	您认为自己现在的体重状况怎么样？	1 偏瘦<br>2 正常<br>3 超重<br>4 肥胖	
F1d	过去 12 个月里，您是否采取过措施控制体重？	1 采取了措施来减轻体重<br>2 采取了措施来保持体重<br>3 采取了措施来增加体重 …….➔<br>4 未采取任何措施 …………….➔	 <br>F2a<br>F2a
F1e	您控制或减轻体重的方法有哪些？（可多选）	1 控制饮食<br>2 锻炼<br>3 药物<br>88 其他	
**F2 血压及其控制**			
F2a	您最近一次测量血压的时间？	1 7 天内<br>2 1 个月内<br>3 6 个月内<br>4 12 个月内<br>5 12 个月以前<br>6 从来没测过血压 …………….➔<br>99 记不清	 <br><br><br><br>F3a
F2b	您是否知道自己的血压情况？	1 高于正常范围<br>2 属于正常范围<br>3 低于正常范围<br>99 不知道	

F2c	您有没有被医生诊断过高血压？	1 有<br>2 没有 ……………………→	F3a
F2d	您被**确诊**高血压的最高级别医疗单位为：	1 省级及以上医院<br>2 地区级（市）医院<br>3 县级（区）医院<br>4 乡镇卫生院（社区卫生服务中心）<br>5 村卫生室（社区卫生服务站、私人诊所）<br>99 不知道	
F2e	您采取了什么措施来控制血压?（**可多选**）	1 未采取任何措施<br>2 按医嘱服药<br>3 有症状时服药<br>4 控制饮食<br>5 运动<br>6 血压监测<br>88 其他	
F2f	最近 2 周，您是否服用了降压药？	1 是<br>2 否	
F2g	您是否参加了基层医疗卫生机构提供的高血压病随访管理？<br>**（指在社区卫生服务中心/站、乡镇卫生院/村卫生室接受定期或不定期检查、治疗、合理膳食和运动等指导）**	1 是<br>2 否…………………………………→<br>99 不知道……………………………→	F3a<br>F3a
F2h	过去 12 个月内，基层医疗卫生机构医生是否为您提供过以下检查或指导？（**可多选**）	1 测量血压，□□□次/年<br>2 用药指导，□□次/年<br>3 饮食指导<br>4 身体活动指导<br>5 戒烟或少吸烟<br>6 戒酒或少饮酒<br>7 上述检查或指导均没有	
**F3 血糖及其控制**			
F3a	您最近一次测量血糖距离现在有多长时间？	1 6 个月内<br>2 12 个月内<br>3 12 个月前<br>4 从来没测过血糖 …………→<br>99 记不清	F4a
F3b	您是否知道自己的血糖情况？	1 高于正常范围<br>2 属于正常范围<br>3 低于正常范围<br>99 不知道	
F3c	您有没有被医生诊断患有糖尿病？<br>**调查员注意：不包括妊娠期糖尿病。**	1 有<br>2 没有 ……………………………→	F4a

F3d	您被**确诊**糖尿病的最高级别医疗单位为：	1 省级及以上医院<br>2 地区级（市）医院<br>3 县级（区）医院<br>4 乡镇卫生院（社区卫生服务中心）<br>5 村卫生室（社区卫生服务站、私人诊所）<br>99 不知道	
F3e	您采取了什么措施来控制血糖？（可多选）	1 未采取任何措施<br>2 口服药<br>3 胰岛素注射<br>4 控制饮食<br>5 运动<br>6 血糖监测<br>88 其他	
F3f	您是否参加了基层医疗卫生机构提供的糖尿病随访管理？<br>**（指在社区卫生服务中心/站、乡镇卫生院/村卫生室接受定期或不定期检查、治疗、合理膳食和运动等指导）**	1 是<br>2 否………..…..………………➔<br>99 不知道…….……..……..…… ➔	 F4a<br>F4a
F3g	过去 12 个月内，基层医疗卫生机构医生是否为您提供过以下检查或指导？（可多选）	1 测量血压，□□□次/年<br>2 测量血糖，□□□次/年<br>3 用药指导，□□次/年<br>4 饮食指导<br>5 身体活动指导<br>6 戒烟或少吸烟<br>7 戒酒或少饮酒<br>8 上述检查或指导均没有	
**F4 血脂及其控制**			
F4a	您最近一次测量血脂距离现在有多长时间？	1 6 个月内<br>2 12 个月内<br>3 12 个月前<br>4 从来没测过血脂 ………….➔<br>99 记不清	F5a
F4b	您有没有被**乡镇卫生院或社区卫生服务中心或以上级别医疗机构**医生诊断为血脂异常或高血脂？	1 有<br>2 没有 …………….……....……➔	F5a
F4c	您采取了什么措施来控制血脂？（可多选）	1 未采取任何措施<br>2 按医嘱服药<br>3 控制饮食<br>4 运动<br>5 血脂监测<br>88 其他	

**F5 心脑血管事件**			
F5a	您是否曾被**县/区级及以上医疗机构**医生诊断为心肌梗死？	1 是<br>2 否……………………………………➔	F5c
F5b	您**首次**确诊为心肌梗死的时间为？<br>哪年哪月<br>或者<br>多大年龄时	□□□□年□□月<br>或<br>□□周岁	
F5c	您是否曾被**县/区级及以上医疗机构**医生诊断为脑卒中？	1 是<br>2 否……………………………………➔	F6a
F5d	您**首次**确诊为脑卒中的时间为？<br>哪年哪月<br>或者<br>多大年龄时	□□□□年□□月<br>或<br>□□周岁	
**F6 其他慢性病**			
F6a	您是否曾被**县/区级及以上医疗机构诊断**为慢性阻塞性肺部疾病（如慢支、肺气肿）？	1 是<br>2 否	
F6b	您是否曾被**县/区级及以上医疗机构诊断**为哮喘？	1 是<br>2 否	
F6c	您是否曾被**县/区级及以上医疗机构诊断**为恶性肿瘤（包括全身恶性肿瘤和颅脑良性肿瘤）？如果有，是什么部位的肿瘤？	1 未被诊断过<br>2 肺癌<br>3 胃癌<br>4 食管癌<br>5 肝癌<br>6 结直肠癌<br>7 乳腺癌<br>8 宫颈癌<br>88 其他	
**F7 呼吸系统状况（仅限 40 岁及以上调查对象），若＜40 岁**		**…………………………➔**	**G1a**
F7a1	过去 12 个月里，早晨您醒来后是否经常咳嗽?	1 是<br>2 否	
F7a2	过去 12 个月里，您是否经常白天或晚上咳嗽?	1 是<br>2 否	
若上述 F7a1、F7a2 两个问题任一个回答为“是”则继续，均回答为“否”则跳至 F7b1			
F7a3	您这样咳嗽是否会每年持续三个月或更长时间?	1 是<br>2 否	
F7b1	过去 12 个月里，您早晨醒来是否有咳痰?	1 是<br>2 否	
F7b2	过去 12 个月里，您是否经常白天或晚上有咳痰?	1 是<br>2 否	
若上述 F7b1、F7b2 个问题任一个回答为“是”则继续，均回答为“否”则跳至 F7c			
F7b3	您这样咳痰是否会每年持续三个月或更长时间?	1 是<br>2 否	
F7c	您有没有做过肺功能检查？	1 是<br>2 否<br>99 不清楚	

第七部分 健康状况

**G1 总体健康状况**			
G1a	总体上看，您认为您的健康状况如何？	1 非常好<br>2 好<br>3 一般<br>4 差<br>5 非常差	
G1b	在过去 30 天里，由于**患病**造成您健康状况不好的天数为？	□□天	
G1c	在过去 30 天里，由于**伤害**造成您健康状况不好的天数为？	□□天	
G1d	在过去 30 天里，由于**紧张、压抑或情绪问题**造成您健康状况不好的天数为？	□□天	
**G2 健康体检**			
G2a	您最近一次进行健康体检距现在多长时间了？（不包括看病时的体检）	1 □□年□□月<br>2 从未体检过 ....➔	 G3
G2b	您健康体检的原因？	1 单位免费提供<br>2 社区免费提供<br>3 自我保健<br>88 其他	
**G3 女性宫颈癌和乳腺癌筛查（仅限女性）**<br>若为男性 ....➔			H1
G3a	您是否接受过宫颈癌筛查？如果接受过，最近一次检查是在什么时候？<br>**调查员注意：不到 1 年填"0"。**	1 有，□□年前<br>2 没有 ....➔<br>99 不清楚 ....➔	 G3c<br>G3c
G3b	您最近一次接受宫颈癌筛查是采用以下哪种方法？	1 细胞学检查（包括传统巴氏涂片和液基细胞学检查）<br>2 人乳头瘤病毒检测<br>3 肉眼观察<br>88 其他方法<br>99 不清楚	
G3c	您是否接受过乳腺癌筛查？如果接受过，最近一次检查是在什么时候？<br>**调查员注意：不到 1 年填"0"。**	1 有，□□年前<br>2 没有 ....➔<br>99 不清楚 ....➔	 H1<br>H1
G3d	您最近一次接受乳腺癌筛查是采用以下哪种方法？	1 乳腺 X 线检查<br>2 乳腺超声检查<br>3 临床检查<br>88 其他方法<br>99 不清楚	

第八部分　口腔卫生

H1	您上一次看牙距现在多长时间？	1　不到 1 年<br>2　1-2 年<br>3　3-4 年<br>4　5 年及以上<br>5　从没看过牙 ……………………➔<br>99　记不清 …..…………….…..➔	 <br><br><br>H3<br>H3
H2	最近一次看牙医的原因是？	1　急性牙疼等口腔问题<br>2　慢性口腔问题去检查或治疗<br>3　接受预防性措施<br>4　定期口腔检查<br>5　寻求牙齿美容治疗<br>88　其他口腔疾病	
	调查员注意：若 H1 选择“1”，跳转到 …………………. ……………………➔		H4
H3	过去 12 个月内，您没有看牙医的主要原因是？	1　牙齿没有问题<br>2　牙病不重<br>3　没有时间<br>4　花费太高，看不起牙<br>5　附近没有牙科诊所或医院<br>6　害怕疼痛<br>7　挂号太难，过程繁琐<br>8　害怕传染病<br>88　其他原因	
H4	您上一次洗牙距现在多长时间？	1　不到 1 年<br>2　1-2 年<br>3　3-4 年<br>4　5 年及以上<br>5　从没洗过 ..……………………➔<br>99　记不清 ……………………➔	 <br><br><br>H6<br>H6
H5	您最近一次洗牙的原因是？	1　治疗疾病　　3　为了外观美观<br>2　预防疾病　　4　去除口臭	
H6	您每天刷牙几次？	1　2 次或以上　　3　不足 1 次<br>2　1 次　　4　不刷牙	
H7	您认为口腔疾病与以下哪些疾病有关系？<br>（可多选）	1　糖尿病<br>2　高血压、心脏病等心血管疾病<br>3　肺炎等呼吸系统疾病<br>4　胃炎等消化系统疾病<br>5　骨质疏松<br>6　早产，低出生体重<br>7　都没有关系<br>99　不知道	
H8	您对自己目前的口腔健康状况如何评价？	1　好<br>2　一般<br>3　差	

第九部分　伤害及其危险因素			
J1	过去 30 天里，您是否乘坐或者驾驶过摩托车？	1　是<br>2　否……………………..……........➔	J3
J2	您乘坐或者驾驶摩托车时，是否有佩戴头盔？	1　总是佩戴<br>2　经常佩戴<br>3　有时佩戴<br>4　很少佩戴<br>5　从不佩戴	
J3	过去 30 天里，您是否乘坐过机动车？	1　是<br>2　否……………………..……........➔	J6
J4	您乘坐机动车时，是否有佩戴安全带？（无论乘坐前排座位或者后排座位）	1　总是佩戴<br>2　经常佩戴<br>3　有时佩戴<br>4　很少佩戴<br>5　从不佩戴<br>6　乘坐的机动车没有安全带	
J5	您乘坐过几次由饮酒后的人所驾驶的机动车？	1　没有<br>2　1 次<br>3　2 或 3 次<br>4　4 或 5 次<br>5　6 次或更多	
J6	过去 30 天里，您是否驾驶过机动车？	1　是<br>2　否……………………..……........➔	结束
J7	您驾驶机动车时，是否有佩戴安全带？	1　总是佩戴<br>2　经常佩戴<br>3　有时佩戴<br>4　很少佩戴<br>5　从不佩戴<br>6　驾驶的机动车没有安全带	
J8	过去 30 天里，您有过几次酒后驾驶机动车的经历？	1　没有<br>2　1 次<br>3　2 或 3 次<br>4　4 或 5 次<br>5　6 次或更多	
J9	过去 30 天里，您是否至少有过一次连续驾驶机动车 4 小时以上，中途没有休息的经历？	1　是<br>2　否	

调查结束时间（24 小时制）：□□ 时 □□ 分

中国慢性病及其危险因素监测（2013）

身体测量记录表

个人编码：□□□□□□□□□□

**身高、体重、腰围询问**		
**您好，下面我们会问您几个关于身高、体重、腰围和血压的问题。**		
K1	您知道自己的身高吗？	1 知道，为□□□.□厘米（cm）<br>99 不知道
K2	您知道自己的体重吗？	1 知道，为□□□.□公斤（kg）<br>99 不知道
K3	您知道自己的腰围吗？	1 知道，为□□□.□厘米（cm）<br>99 不知道
**身体测量**		
**您好，下面我们将测量您的身高、体重、腰围和血压，请您配合。**		
M1a	测量员姓名 1	________________
M1b	测量员姓名 2	________________
M2	身高<br>调查员注意：身高如果超过量程，记录-9。	□□□.□厘米（cm）
M3	体重<br>调查员注意：体重如果超过量程，记录-9。	□□□.□公斤（kg）
**腰围**		
M4	腰围	□□□.□厘米（cm）

血压和心率			
M5	室内温度	□□.□ ℃	
M6	测量员姓名	________________	
M7a	第 1 次读数<br>**调查员注意：测量对象休息 5 分钟后第 1 次测量并记录血压，休息 1 分钟后第 2 次测量血压和心率**	收缩压	□□□ mmHg
M7b		舒张压	□□□ mmHg
M7c		心率	□□□ 次/分
M8a	第 2 次读数<br>**调查员注意：记录第 2 次测量结果，待测量对象再休息 1 分钟后第 3 次测量血压和心率**	收缩压	□□□ mmHg
M8b		舒张压	□□□ mmHg
M8c		心率	□□□ 次/分
M9a	第 3 次读数<br>**记录第 3 次测量结果**	收缩压	□□□ mmHg
M9b		舒张压	□□□ mmHg
M9c		心率	□□□ 次/分

中国慢性病及其危险因素监测（2013）

口腔健康检查记录表

个人编码：□□□□□□□□□□

N1	牙列状况	18 17 16 15 14 13 12 11 21 22 23 24 25 26 27 28<br>□□□□□□□□□□□□□□□□<br>48 47 46 45 44 43 42 41 31 32 33 34 35 36 37 38<br>□□□□□□□□□□□□□□□□
	牙列状况符号	0 无龋<br>1 有龋<br>2 已填充有龋<br>3 已充填无龋<br>4 因龋缺失<br>5 因其它原因缺失<br>6 未萌牙<br>7 牙外伤<br>8 其他或无法检查
N2	牙周状况（CPI）	0 健康<br>1 牙龈出血<br>2 牙石（无牙龈出血）<br>3 牙石（有牙龈出血）<br>4 浅牙周袋<br>5 深牙周袋<br>X 除外区段（少于两颗功能牙存在）<br>9 无法检查<br>16/17 11 26/27<br>□ □ □<br>□ □ □<br>46/47 31 36/37
N3	义齿修复状况	□<br>0 不需要修复<br>1 完全未修复<br>2 部分修复<br>3 全部修复
