# Supplementary material for: Comparative analysis of IDF, ATPIII and CDS in the diagnosis of metabolic syndrome among adult inhabitants in Jiangxi Province, China
Source: PLoS One. 2017 Dec 7;12(12):e0189046. doi: 10.1371/journal.pone.0189046 (PMC5720703; doi:10.1371/journal.pone.0189046)
Supplement: S9 Table — (DOCX) [file pone.0189046.s009.docx]

**Paste the questionnaire**

**Whether diabetes：1 Yes 2 No**

According to the People's Republic of China "Statistical Law" Chapter III of Article XV provisions,”Single item investigation data concerning any individual or his/her family shall not be divulged without the consent of the said person.”

**2013 NCD Surveillance**

**Individual Questionnaire**

| Name：_______________ Telephone__________________________ | |
| --- | --- |
| Monitoring area(county/area):__________ | Monitoring area code: |
| Township / Street name:__________ | Township / Street code: |
| Village / Neighborhood committees name:__________ | Village / Neighborhood committees code: |
| Family code: | |
| Investigator’s signature:__________ | Date: / / |
| Monitoring points quality control personnel’s signature:__________ | Date: / / |
| Provincial supervisor’s signature:__________ | Date: / / |

**Chinese Center for Disease Control and Prevention**

**National Center for Chronic Noncommunicable Disease Control and Prevention**

**JUNE 2013**

**Paste the blood collection code**

**Survey start time(24 hours systems):**

| Section 1 Basic Information | | | | |
| --- | --- | --- | --- | --- |
| A1 | Date of birth  **Investigator Note: which can not remember in the corresponding items by right fill "-9"** | (dd/mm/yyyy) | |  |
| A2 | Gender | 1. Male 2. Female | |  |
| A3 | Ethnic group | 1. Han 2. Zhuang 3. Man 4. Hui 5. Miao 6. Uighur | 1. Yi 2. Tujia 3. Mongol 4. Korean 5. Tibet   88 Others |  |
| A4 | Highest level of education | 1. No formal school 2. <primary school 3. Primary school 4. Secondary school 5. High school/secondary school/   technical school   1. College 2. University 3. Postgraduate and above | |  |
| A5 | Marital status | 1. Single 2. Married 3. Cohabited | 1. Widowed 2. Divorced 3. Separated |  |
| A6 | Occupation | 1. Agriculture, fishery and related labourers 2. Plant and machine operators and assemblers 3. Sales and services elementary occupations 4. State organs, party organizations, enterprises, institutions responsible person 5. Staff and personnel concerned 6. Professional skill worker 7. Military 8. Others 9. Students 10. Unemployed 11. Housework 12. Retired | |  |
| A7 | What kind of medical insurance do you have? **(Multiple choice)**  **Investigator Note: please read** | 1. Basic medical insurance 2. Medical services at state expense 3. Medical insurance for urban and township resident 4. New rural cooperative medical insurance 5. Commercial medical insurance 6. Others 7. No medical insurance   99 Don’t know/not sure | |  |
| A8 | What is your Domicile? | 1. The county (district) 2. In the rest of the city 3. Other counties in the local city 4. In the province (autonomous regions   or municipalities) owned by other cities   1. In other provinces (autonomous regions   or municipalities) | |  |

| Section 2 Tobacco Use | | | |
| --- | --- | --- | --- |
| **Current Smoking** | | | |
| B1 | Do you currently smoke tobacco on a daily basis, less than daily or not at all? | 1. Yes, daily 2. yes, less than daily……................……..…🡺 3. Before,not now……………..…................…🡺 4. Not at all ………………...............…..……🡺 | B3  B8  B11 |
| B2 | When did you start smoking every day?  **Investigator Note: Can not remember fill "-9"** | ____years |  |
| B3 | How much do you smoke cigarettes on average each day/week?  **Investigator Note: Daily smokers answer option 1, non-daily smokers answer option 2** | 1. ____/day 2. ____/week 3. Do not smoke cigarettes | |
| **Cessation** | | | |
| B4 | Did you quit smoking in the past?（quit smoking means consider seriously and had some actions to quit） | 1 Yes,over the past 12 months  2 Yes,12 months ago…..………..............…....🡺  3 No ……..…………..………..........…........🡺 | B6  B6 |
| B5 | In the past 12 months, have you tried to quit smoking by nicotine replacement therapy or other western drugs? | 1 yes  2 no | |
| B6 | Which of the following was your thought on quitting smoking? | 1 attempt to quit in a month  2 attempt to quit in 12 months  3 attempt to，but not within 12 months  4 don’t want to quit  99 don’t know | |
| B7 | Has the doctor advised you to quit smoking when you see the doctor in the past 12 months? | 1 Have not seen a doctor……..............…....🡺  2 The doctor had suggested smoking cessatio🡺  3 The doctor do not recommend smoking cessation….....…...........…..............…..............…....🡺 | B11  B11  B11 |
| B8 | How long has it been since you stop smoking?  Investigators Note: Only include the respondent completely quit smoking, and the occasional smoking is not included. Note that only one can be filled out. | a ____years  b ____months  c ____weeks  d ____days | |
|  | **Investigators Note: If B8<1year（<12months） .....……...…………......…......…...…🡺**  **Otherwise …….......…......…......……...………….......…......…....…...….…..…...…🡺** | | B9  B11 |
| B9 | Has the doctor advised you to quit smoking when you see the doctor in the past 12 months? | 1 Have not seen a doctor  2 The doctor had suggested smoking cessation  3 The doctor do not recommend smoking cessation | |
| B10 | In the past 12 months, have you tried to quit smoking by nicotine replacement therapy or other western drugs? | 1. Yes 2. no | |
| **Passive Smoking** | | | |
| B11 | Generally, how many days are you exposed to second hand smoke every week? | 1. every day 2. 4-6 days a week 3. 1-3 days a week 4. never   2 not exposed | |

| **Knowledge, Attitude And Understanding** | | | | | | |
| --- | --- | --- | --- | --- | --- | --- |
| B12 | As far as you know, will smoking cause very serious illness? | | 1. Yes 2. No ..…......…......….....…🡺   99 don’t know ..…......…......…...…🡺 | | | B14  B14 |
| B13 As far as you know, will smoking cause the following diseases? | | | | | | |
|  | | yes | | no | Don’t know/not sure | |
| a | Stroke (stroke, cerebral thrombosis) | 1 | | 2 | 99 | |
| b | heart attack | 1 | | 2 | 99 | |
| c | Lung cancer | 1 | | 2 | 99 | |
| B14 | As far as you know, inhalation of secondhand smoke will cause serious illness? | | 1 Yes  2 No ..…......…......….....…🡺  99 don’t know ..…......…......…...…🡺 | | | B16  B16 |
| B15 As far as you know, inhalation of secondhand smoke will cause the following diseases? | | | | | | |
|  | | yes | | no | Don’t know/not sure | |
| a | Adult heart disease | 1 | | 2 | 99 | |
| b | Children with lung disease | 1 | | 2 | 99 | |
| c | Adult lung cancer | 1 | | 2 | 99 | |
| B16 | Do you agree to mark that the low tar content cigarettes is less harmful than cigarettes? | | 1. agree 2. disagree   99 don’t know | | | |

| Section 3 Drinking Situation | | | | | | | | | | |
| --- | --- | --- | --- | --- | --- | --- | --- | --- | --- | --- |
| C1 | | Have you consumed any alcohol within the past 12 months? | | 1 Yes，in the past 30 days  2 Yes，before the past 30 days  3 No ……………………………🡺 | | | | D1 | | |
| C2 | | In the past 12 months, how frequently have you had alcoholic beverages?  **(Investigators Note:Need to read options)** | | 1 Everyday  2 5-6days/week  3 3-4days/week  4 1-2days/week  5 1-3day/month  6 <1day/month | | | | | | |
| Please answer:In the past 12 months, how many days per week or per month or per year did you have the following alcoholic beverage and on the days when you drank, how many drinks did you drink per day on the average.  **Investigators Note:Can not remember in the decimal point before the right fill "-9", no drinking will not fill the frequency of drinking and drinking.** | | | | | | | | | | |
| C3 |  | | a drink or not  1yes，2no | b frequency（fill one column only） | | | amount of drinking on average on the days when you drank | | |  |
|  |  |  |  | b1  day/week | b2  day/month | b3  day/year |  |  |  |  |
|  | a. white wine（≥42%vol） | |  |  |  |  | .liang | | |  |
|  | b. white wine（<42%vol） | |  |  |  |  | .liang | | |  |
|  | c. beer(580ml/bottle,4%vol  ) | |  |  |  |  | .bottles | | |  |
|  | d. yellow wine(18%vol) | |  |  |  |  | .liang | | |  |
|  | e. rice wine(18%vol) | |  |  |  |  | .liang | | |  |
|  | f. red wine(10%vol) | |  |  |  |  |  | | |  |
|  | g.highland barley wine(13%vol) | |  |  |  |  | .liang | | |  |
| C4 | **a. For males**：  In the past 12 months，how frequent did you have 2.5liang high alcohol white wine, or 3.5liang low alcohol white wine, or 3 bottles of beer, or 7.5liang yellow wine or 1.5jin red wine or more on one occasion? | | | 1 Daily or almost daily(≥5days/week)  2 1-4days/week  3 1-3days/month  4 ＜1day/month  5 Never | | | | |  |  |
|  | **b. For females：**  In the past 12 months，how frequent did you have 2liang high alcohol white wine, or 3liang low alcohol white wine, or 2.5 bottles of beer, or 6liang yellow wine or 1.2jin red wine or more on one occasion? | | | 1 Daily or almost daily(≥5days/week)  2 1-4days/week  3 1-3days/month  4 ＜1day/month  5 Never | | | | |  |  |

| Section 4 Diet | | | | |
| --- | --- | --- | --- | --- |
| D1 | In the past 12 months, how many meals do you have every day on average? | _____meals | | |
|  | | Place of having meals | | |
|  |  | A home | B Canteen | C restaurant |
| D2 | In the past 12 months，how many days do you have breakfast in the different places in a typical week? |  days |  days |  days |
| D3 | In the past 12 months，how many days do you have lunch in the different places in a typical week? |  days |  days |  days |
| D4 | In the past 12 months，how many days do you have supper in the different places in a typical week? |  days |  days |  days |

| Please remember，in the past 12 months, did you have the listed food? and if yes, please estimate the frequency of eating and the amount of eating every time? | | | | | | | |
| --- | --- | --- | --- | --- | --- | --- | --- |
|  | | a eat or not  1yes，2no | b frequency（fill one column only） | | | | amount of intake every time |
|  |  |  | b1  times/day | b2  times/week | b3  times/month | b4  times/year |  |
| D5 | Pork |  |  |  |  |  | .liang |
| D6 | Lamb and beef |  |  |  |  |  | .liang |
| D7 | Poultry |  |  |  |  |  | .liang |
| D8 | Aquatic products |  |  |  |  |  | .liang |
| D9 | Fresh vegetables |  |  |  |  |  | .liang |
| D10 | Fresh fruits |  |  |  |  |  | .liang |
| D11 | Carbonated drinks  (250ml/glass) |  |  |  |  |  |  glasses |
| D12 | Fruit juice / fruit drinks(250ml/glass) |  |  |  |  |  |  |

| D13 | Do you know that Chinese residents dietary guidelines recommend adults to eat salt every day should not exceed a few grams? | 1. Yes, ____gram   88 don’t know | |
| --- | --- | --- | --- |
| D14 | Which of the following diseases do you think could be caused by eating too much salt **?**  **(multiple choices)** | 1. Hypertension 2. Stroke 3. Myocardial infarction   4 kidney disease | 5 No association  88 Others  99 Not clear |
| D15 | Do you think you eat too much salt? | - 1. Less   2. Moderate   3. Excessive   99 Not clear | |
| D16 | If you know that eating salt is harmful to health, are you willing to eat less? | 1 willing  2 Do not want to  3 It does not matter  4 Not clear | |
| D17 | Have you taken any salt reduction measures? If so, what measures have you taken？  **(multiple choices)** | 1 Did not take any salt reduction measures  2 Reduce eating out  3 Cooking food less salt  4 Eat high salt foods such as pickled foods, fermented bean 5 curd, salted duck eggs, miso,  6 Do not add any extra salt when you eat at the table  7 Use salt-limited tools such as salt spoons  8 Use low sodium salt  9 other | |

| Section5 Physical Activity | | | |
| --- | --- | --- | --- |
| The following questions are usually a week when you carry out various physical activities (including dry farming, work, housework, traffic related physical activity, recreational exercise or exercise). Please answer: | | | |
| **Work, Agriculture And Housework** | | | |
| E1 | Are there any high-intensity sports for at least 10 minutes continuously in your work, farm work and housework？  (High intensity activity means that causes large increases in breathing or heart rate like carrying or lifting heavy loads, digging or construction work)  Investigators Note: The identity activity table can be presented. | 1 Yes  2 No ………………….............…🡺 | E4 |
| E2 | How many days of your week will be done high-intensity activities in your work, farm work and housework？ | _____days | |
| E3 | How much time do you spend doing vigorous-intensity activities at work on a typical day in your work, farm work and housework?  Investigators Note: If the time of each event is less than 10 minutes, it is not included. | _____hours_____minutes | |
| E4 | Does your work , farm work and housework have the moderate-intensity activity for at least 10 minutes continuously?  (Moderate-intensity activity means sawing wood, washing clothes, cleaning and other needs to pay moderate physical strength, or cause breathing, heartbeat slightly increased activities.)  Investigators Note: The identity activity table can be presented. | 1. Yes   2 No ……………………..........🡺 | E7 |
| E5 | How many days of your week will be done moderate-intensity activities in your work, farm work and housework？ |  _____days | |
| E6 | How much time do you spend doing moderate-intensity activities at work on a typical day in your work, farm work and housework? | _____hours_____minutes | |

| **Traffic Physical Activity**  The following questions do not include the above mentioned agricultural physical activity and work and household physical activity. | | | | | | |
| --- | --- | --- | --- | --- | --- | --- |
| E7 | Do you walk or use a bicycle for at least 10 minutes continuously to get to and from places? | | | 1 Yes  2 No ……………………...........🡺 | | E10 |
| E8 | In a typical week, on how many days do you walk or bicycle for at least 10 minutes continuously to get to and from places? | | |  _____days | |  |
| E9 | How much time do you spend walking or bicycling for travel on a typical day? | | | _____hours_____minutes | |  |
| **Recreational Activities**  The following questions do not include the above mentioned agricultural, work, household and traffic physical activities. | | | | | | |
| E10 | | Do you do any vigorous-intensity sports that cause large increases in breathing or heart rate for at least 10 minutes continuously? Such as long-distance running, swimming, playing football and so on.  Investigators Note: The identity activity table can be presented. | | 1 Yes  2 No …………………….........🡺 | | E13 |
| E11 | | In a typical week, on how many days do you do vigorous-intensity sports, fitness or recreational activities? | |  _____days | |  |
| E12 | | How much time do you spend doing vigorous-intensity sports, fitness or recreational activities on a typical day? | | _____hours_____minutes | |  |
| E13 | | Do you do any moderate-intensity sports or recreational activities that cause a small increase in breathing or heart rate for at least 10 minutes continuously? | | 1 Yes  2 No ……………..……......🡺 | | E16 |
| E14 | | In a typical week, on how many days do you do moderate-intensity sports, fitness or recreational activities? | | _____days | |  |
| E15 | | How much time do you spend doing moderate-intensity sports, fitness or recreational activities on a typical day? | | _____hours_____minutes | |  |
| **Total Static Behavior** | | | | | | |
| E16 | | How much time do you usually spend sitting or reclining on a typical day?(Including sitting, working, reading, watching TV, using computer, resting all the time of static behavior, but not including sleeping time.) | | _____hours_____minutes |  | |
| **Amateur Time Static Behavior** | | | | | | |
| E17a | | In spare time，How much time do you spend on watching TV every day? | | _____hours_____minutes | | |
| E17b | | In spare time，how many hours do you spend on the internet every day?(Including desktop computers, laptops, tablet PCs and more.) | | _____hours_____minutes | | |
| E17c | | In spare time，How much time do you spend on mobile phone every day? | | _____hours_____minutes | | |
| E17d | | In spare time，How much time do you spend on reading (paper reading) every day? | | _____hours_____minutes | | |
| **Sleep** | | | | | | |
| E18 | | | In spare time，how many hours do you sleep every day? | _____hours_____minutes |  | |

| **Section 6 Weight, Blood Pressure, Blood Glucose and Lipid** | | | | | | |
| --- | --- | --- | --- | --- | --- | --- |
| **F1 Weight And Its Control** | | | | | | |
| F1a | When was the last time you measured weight? | 1. Never 2. Within 7 days 3. Within the last month 4. Within the last 3 months 5. Within the last 6 months 6. Within the last 12 months 7. 12 months ago   99 Not sure/Don’t know | | | | |
| F1b | Do you have any changes in weight compared with 12 months ago? | 1. Increased by 2.5 kg or more 2. Basically remain unchanged (increase or decrease within 2.5 kg) 3. Fell more than 2.5 kg   99 Not sure/Don’t know | | | | |
| F1c | What do you think of your current weight now? | 1 Lean  2 Normal  3 Overweight  4 Obesity | | | |  |
| F1d | In the past 12 months, did you take any measures to control weight? | 1. Measures to lose weight 2. Measures to keep current weight 3. Measures to increase weight ………........🡺 4. No ………………...........…🡺 | | | | F2a  F2a |
| F1e | What measures have you taken to lose or control your weight? **(multiple choices）** | 1. Control diet 2. Exercises 3. Medication   88 Others | | | | |
| **F2 Blood Pressure And Its Control** | | | | | | |
| F2a | When was the last time you measured blood pressure? | 1 Within 7 days  2 Within the last month  3 1-6 months ago  4 1-12 months ago  5 12 months ago  6 Ever ………………...…............🡺  99 Not sure/don’t know | | | F3a | |
| F2b | Do you know your blood pressure? | 1. Higher than normal 2. Belong to normal circumstances 3. Lower than normal   99 Don’t know | | | | |
| F2c | Have you been diagnosed to have hypertension？ | 1 Yes  2 No …………………......................................🡺 | | | F3a | |
| F2d | What is the highest level of medical units that you diagnosed high blood pressure？ | 1. Provincial and above hospitals 2. Regional (city) hospital 3. County (district) hospital 4. Township Health Center (Community Health Service Center) 5. Village health room (community health service station, private clinic)   99 Don’t know | | | | |
| F2e | What kind of measures did you take  to control your blood pressure?  **（multiple choices）** | 1. Did not take any measures 2. Take drugs according to doctor’s prescription 3. Take drugs when symptoms present 4. Control diet 5. Exercises 6. Blood pressure monitoring   88 Others | | | | |
| F2f | Did you take blood pressure lowering medication in the past 2 weeks? | 1. Yes 2. No | | | | |
| F2g | Did you participate in the follow - up management of hypertension at the Primary health care institutions?  (Means to guidance at the community health service center / station, township health center / village health room for regular or irregular inspection, treatment, reasonable diet and exercise) | 1 Yes  2 No ………………...…............🡺  99 Don’t know ………………...............…🡺 | | | F3a  F3a | |
| F2h | Has the doctors at primary health care institutions provided you with the following checks or guidance over the past 12 months?  （multiple choices） | 1. Measure blood pressure, ____times/year 2. Guidance on medication, ____times/year 3. Dietary guidance 4. Guidance for physical activity 5. Quit smoking or less smoking 6. Quit smoking or less alcohol 7. None of the above checks or instructions | | | | |
| **F3 Blood Glucose And Its Control** | | | | | | |
| F3a | How long has it been since you last measured your blood glucose? | 1. 1-6 months 2. 7-12 months 3. >12months 4. Never measured …......................🡺   99 Not sure/Don’t know | | | F4a | |
| F3b | Do you know your blood glucose situation? | 1 Higher than normal  2 Belong to normal circumstances  3 Lower than normal  99 Don’t know | | | | |
| F3c | Have you been diagnosed to have diabetes？  **Investigators Note: not including gestational diabetes** | 1. Yes   2 No ............................…..………....🡺 | | | F4a | |
| F3d | What is the highest level of medical units that you diagnosed diabetes？ | 1. Provincial and above hospitals 2. Regional (city) hospital 3. County (district) hospital 4. Township Health Center (Community Health Service Center) 5. Village health room (community health service station, private clinic)   99 Don’t know | | | | |
| F3e | What kind of measures did you take  to control your blood glucose?  **（multiple choices）** | 1. Did not take any measures 2. Take drugs according to doctor’s prescription 3. Insulin treatment 4. Control diet   4 Exercises  5 Blood glucose monitoring  88 Others | | | | |
| F3f | Did you participate in the follow - up management of diabetes at the Primary health care institutions?  (Means to guidance at the community health service center / station, township health center / village health room for regular or irregular inspection, treatment, reasonable diet and exercise) | 1 Yes  2 No ………………...….............🡺  99 Don’t know ………………................…🡺 | | | F4a  F4a | |
| F3g | Has the doctors at primary health care institutions provided you with the following checks or guidance over the past 12 months?  **（multiple choices）** | 1. Measure blood pressure, ____times/year 2. Measure blood glucose, ____times/year 3. Guidance on medication, ____times/year 4. Dietary guidance 5. Guidance for physical activity 6. Quit smoking or less smoking 7. Quit smoking or less alcohol 8. None of the above checks or instructions | | | | |
| **F4 Blood Lipids And Its Control** | | | | | | |
| F4a | How long has it been since you last measured your blood lipids? | 1 1-6 months  2 7-12 months  3 >12months  4 Never measured …....................🡺  99 Not sure/Don’t know | | | F5a | |
| F4b | Have you been diagnosed as dyslipidemia or hyperlipidemia by a doctor in a township health center or community health service center or above? | 1 Yes  2 No ........................……..………...🡺 | | | F5a | |
| F4c | What kind of measures did you take  to control your blood lipids?  **（multiple choices）** | 1. Did not take any measures 2. Take drugs according to doctor’s prescription 3. Control diet 4. Exercises 5. Blood lipids monitoring   88 Others | | | | |
| **F5 Cardio-cerebrovascular Events** | | | | | | |
| F5a | Have you ever been diagnosed with a myocardial infarction by a doctor at a county / district level or above? | 1 Yes  2 No ………..................................🡺 | | | F5c | |
| F5b | What the time you diagnosed as myocardial infarction at first？  which year which month  or  how old is you age | mm/yyyy  or  _____years | | | | |
| F5c | Have you ever been diagnosed with stroke by a doctor at a county / district level or above? | 1 Yes  2 No ………...................................🡺 | | | F6a | |
| F5d | What the time you diagnosed as stroke at first？  which year which month  or  how old is you age | mm/yyyy  or  _____years | | | | |
| **F6 Other Chronic Diseases** | | | | | | |
| F6a | Have you been diagnosed to have chronic obstructive pulmonary diseases(like chronic bronchitis or emphysema) by a doctor at a county / district level or above? | 1 Yes  2 No | | | | |
| F6b | Have you been diagnosed to have asthma by a doctor at a county / district level or above? | 1 Yes  2 No | | | | |
| F6c | Have you been diagnosed to have malignant tumor(Including systemic malignancy and benign brain tumors)by a doctor at a county / district level or above? | 1. Not diagnosed 2. Lung cancer 3. Stomach cancer 4. Esophageal cancer 5. Liver cancer | 1. Colorectal cancer 2. Breast cancer 3. Cervical cancer 4. other | | | |
| **F7 Respiratory System Status(the survey object only 40 years old or older),**  **If less than 40 years old**  ………...............................🡺 | | | | **G1a** | | |
| F7a1 | Have you ever coughed after you wake up in the past 12 months? | 1 Yes  2 No | | | | |
| F7a2 | Have you often coughed during the day or night in the past 12 months? | 1 Yes  2 No | | | | |
| **If the above F7a1, F7a2 two questions any one answer is "yes" then continue to answer are "no" then jump to F7b1** | | | | | | |
| F7a3 | Have you ever been coughing for three months or more each year? | 1 Yes  2 No | | | | |
| F7b1 | Have you ever had sputum after waking up in the past 12 months? | 1 Yes  2 No | | | | |
| F7b2 | Have you ever had sputum during the day or night in the past 12 months? | 1 Yes  2 No | | | | |
| **If the above F7b1, F7b2 two questions any one answer is "yes" then continue to answer are "no" then jump to F7c** | | | | | | |
| F7b3 | Have you ever been so spit for three months or more each year? | 1 Yes  2 No | | | | |
| F7c | Have you ever done a lung function check? | 1 Yes  2 No  99 Don’t know | | | | |

| Section 7 Health Status | | | | |
| --- | --- | --- | --- | --- |
| **G1 General Health Status** | | | | |
| G1a | In general, how would you describe your health? | | 1. Very good 2. Good 3. Fair 4. Poor   5 Very poor | |
| G1b | Now thinking about your physical health, for how many days during the past 30 days was your physical health not good due to physical illness? | | _____days | |
| G1c | Now thinking about your physical health, for how many days during the past 30 days was your physical health not good due to injury? | | _____days | |
| G1d | Now thinking about your mental health, which includes stress, depression and problems with emotions, for how many days during the past 30 days was your mental health not good? | | _____days | |
| **G2 Health Check Up** | | | | |
| G2a | How long has it been since your last health checkup？(not including doctor consultation when ill) | 1 _____years and _____months  2 Never .....................................🡺 | | G3 |
| G2b | What was the reason for health check up？ | 1 Provided free by my employer  2 Provided free by community  3 Self health care  88 Others | | |
| **G3 Breast and Cervical Cancer Screening （for female only）**  if males,.......................................................................................................................🡺 | | | | H1 |
| G3a | Have you ever had a Pap test？How long has it been since you had your last Pap test？  (Investigators Note: Fill ”0” if <1 year) | 1 Yes, _____years ago  2 No …………………..............…🡺  99 Don’t know/Not sure .........…..........…🡺 | | G3c  G3c |
| G3b | Which method do you sample for the last time you are receiving cervical cancer screening? | 1. Cytology(including Pap smear and TCT) 2. Human papillomavirus detection 3. Visual observation   88 Other methods  99 Not clear | | |
| G3c | Have you ever had a breast cancer screening？How long has it been since you had your last breast cancer screening？  (Investigators Note: Fill ”0” if <1 year) | 1 Yes, _____years ago  2 No …………………...................…🡺  99 Don’t know/Not sure .........…..............…🡺 | | H1  H1 |
| G3d | Which method do you sample for the last time you are receiving breast cancer screening? | 1. Mammography 2. Breast ultrasound 3. Clinical examination 4. Other methods 5. Not clear | | |

| **Section 8 Oral Health** | | | |
| --- | --- | --- | --- |
| H1 | How long has it been since you last visited a dentist? | 1. <1year 2. 1-2years 3. 3-4years 4. >5years 5. Never ........…..........…🡺   99 Not sure ........…..........…🡺 | H3  H3 |
| H2 | What was the reason you last visited a dentist? | 1. Acute toothache and other oral problems 2. Chronic oral problems to check or treat 3. Accept preventive measures 4. Regular oral examination 5. Seeking dental cosmetic treatment 6. Other oral diseases | |
|  | Investigators Note: If H1 selects “1”, then jump to .......................................,.…..........…🡺 | | H4 |
| H3 | What is the main reason you did not see a dentist in the last 12 months? | 1. Teeth no problem 2. The tooth problem is not serious 3. No time 4. It is too expensive to spend 5. There is no dental clinic or hospital nearby 6. Fear of pain 7. Registration is too difficult, the process cumbersome 8. Fear of infectious disease 9. other reasons | |
| H4 | How long has it been since you last had your teeth cleaned ? | 1 <1year  2 1-2years  3 3-4years  4 >5years  5 Never ........…..........…🡺  99 Not sure ........…..........…🡺 | H6  H6 |
| H5 | What was the reason you last had your teeth cleaned ? | 1. Curing disease 2. prevent disease 3. In order to look beautiful 4. Remove bad breath | |
| H6 | How many times do you brush your teeth every day? | 1. 2 times or more 2. 1 time 3. Less than once 4. Do not brush your teeth | |
| H7 | Which of the following diseases do you think are related to oral diseases?  (multiple choices) | 1. Diabetes 2. High blood pressure, heart disease and other cardiovascular diseases 3. Pneumonia and other respiratory diseases 4. Gastritis and other digestive diseases 5. Osteoporosis 6. Premature birth, low birth weight 7. It does not matter 8. do not know | |
| H8 | How do you evaluate your current oral health status? | 1. It is good 2. General 3. Not good | |

| **Section 9 Injury and Risk Factors** | | | | | |
| --- | --- | --- | --- | --- | --- |
| J1 | Did you ride or drive a motorcycle In the past 30 days？ | | 1. Yes 2. No ........…..........…,.................🡺 | | J3 |
| J2 | Did you wear helmet when you rode or took motorcycle? | | 1. Always 2. Often   2 Sometime  3 Very few  4 Never | | |
| J3 | In the past 30 days, have you ever taken a motor vehicle?? | | 1 Yes  2 No ……………….……..……,,,........🡺 | | J6 |
| J4 | In the past 30 days, did you fasten seat belt when you taken motor vehicle?(Regardless of the front seat or rear seat.) | | 1 Always  2 Often  3 Sometime  4 Very few  5 Never  6 The motor vehicle does not have a seat belt | | |
| J5 | How many times have you taken a motor vehicle driven by a drinker? | | 1. Never 2. 1 time 3. 2 or 3 times 4. 4 or 5 times 5. 6 times or more | | |
| J6 | Did you drive a motor vehicle in the past 30 days? | | 1 Yes  2 no …………….………...….🡺 | End | |
| J7 | Did you wear a seat belt when you were driving a motor vehicle? | | 1 Always  2 Often  3 Sometime  4 Very few  5 Never  6 The motor vehicle does not have a seat belt | | |
| J8 | How many times you had drunk driving In the past 30 days? | 1 Never  2 1 time  3 2 or 3 times  4 4 or 5 times  5 6 times or more | | | |
| J9 | In the past 30 days, did you drive for more than 4 hours without rest at least once? | 1 Yes  2 No | | | |

**Survey end time (24 hours systems):**

**2013 NCD Surveillance**

**Body Measurement Record**

个人编码：**□□□□□□□□□□**

| **Height, Weight, Waist Asked** | | | |
| --- | --- | --- | --- |
| **Hello, here we will ask you a few questions about height, weight, waist circumference and blood pressure.** | | | |
| K1 | Do you know your height? | 1 Yes, it is _____cm  99 do not know | |
| K2 | Do you know your weight? | 1 Yes, it is _____kg  99 do not know | |
| K3 | Do you know your waist circumference? | 1 Yes, it is _____cm  99 do not know | |
| **Body Measurement** | | | |
| **Hello, then we will measure your height,weight,waist circumference and blood pressure,please cooperate.** | | | |
| M1a | Name of surveyor1 | _____________ | |
| M1b | Name of surveyor2 | _____________ | |
| M2 | Height  Investigators Note:If the height exceeds the range, record -9 | _________cm | |
| M3 | Weight  Investigators Note:If the height exceeds the range, record -9 | _________kg | |
| **Waist Circumference** | | | |
| M4 | Waist circumference | _________cm | |
| **Blood Pressure And Heart Rate** | | | |
| M5 | Room temperature | _________℃ | |
| M6 | Name of surveyor | _____________ | |
| M7a | First reading  The surveyor note:Measure and record blood pressure for the first time after subjects rest for 5 minutes. Measure for the 2^nd^ time after 1 minute’s rest of the subjects. | SBP | _________(mmHg) |
| M7b |  | DBP | _________(mmHg) |
| M7c |  | HR | _________/minute |
| M8a | second reading  The surveyor note:Record the 2^nd^ reading, measure for the 3^rd^ time after 1 minute’s rest of the subjects. | SBP | _________(mmHg) |
| M8b |  | DBP | _________(mmHg) |
| M8c |  | HR | _________/minute |
| M9a | third reading  (record the 3^rd^ reading) | SBP | _________(mmHg) |
| M9b |  | DBP | _________(mmHg) |
| M9c |  | HR | _________/minute |

**2013 NCD Surveillance**

**Oral Health Checklist**

个人编码：**□□□□□□□□□□**

| N1 | Dentition status 18 17 16 15 14 13 12 11 21 22 23 24 25 26 27 28   \|  \|  \|  \|  \|  \|  \|  \|  \|  \|  \|  \|  \|  \|  \|  \|  \| \| --- \| --- \| --- \| --- \| --- \| --- \| --- \| --- \| --- \| --- \| --- \| --- \| --- \| --- \| --- \| --- \|      \|  \|  \|  \|  \|  \|  \|  \|  \|  \|  \|  \|  \|  \|  \|  \|  \| \| --- \| --- \| --- \| --- \| --- \| --- \| --- \| --- \| --- \| --- \| --- \| --- \| --- \| --- \| --- \| --- \|   48 47 46 45 44 43 42 41 31 32 33 34 35 36 37 38  Dentition status symbol  0 No caries 5 Due to other circumstances missing  1 Caries 6 Not budding yet  2 Filled teeth have caries 7 Tooth trauma  3 Filled teeth without dental caries 8 Other or can not be checked  4 Due to caries loss | | |
| --- | --- | --- | --- | --- | --- | --- | --- | --- | --- | --- | --- | --- | --- | --- | --- | --- | --- | --- | --- | --- | --- | --- | --- | --- | --- | --- | --- | --- | --- | --- | --- | --- | --- | --- | --- |
| N2 | community periodontal index  (CPI) | 0 = gingival health  1 = bleeding gums  2 = calculus, gums without bleeding  3 = calculus, bleeding with gums  4 = shallow periodontal pocket  5 = deep periodontal pocket  X = excluded area, less than two functional teeth present  9 = can not be checked | 16/17 11 26/27   \|  \|  \|  \|  \|  \| \| --- \| --- \| --- \| --- \| --- \| \|  \|  \|  \|  \|  \| \|  \|  \|  \|  \|  \|     46/47 31 36/37 |
| N3 | \|  \| \| --- \|   Denture repair condition  0 Do not need to fix  1 Completely not repaired  2 Partial repair  3 All repair | | |
